# The Shape of Phylogenies Under Phase-Type Distributed Times to Speciation and Extinction

**DOI:** 10.1007/s11538-022-01072-w

**Published:** 2022-09-14

**Authors:** Albert Ch. Soewongsono, Barbara R. Holland, Małgorzata M. O’Reilly

**Affiliations:** School of Natural Sciences (Discipline of Mathematics), University of Tasmania, Hobart, 7005 Australia

**Keywords:** Macro-evolutionary model, Diversification, Tree balance, Phase-type distribution

## Abstract

Phylogenetic trees describe relationships between extant species, but beyond that their shape and their relative branch lengths can provide information on broader evolutionary processes of speciation and extinction. However, currently many of the most widely used macro-evolutionary models make predictions about the shapes of phylogenetic trees that differ considerably from what is observed in empirical phylogenies. Here, we propose a flexible and biologically plausible macroevolutionary model for phylogenetic trees where times to speciation or extinction events are drawn from a Coxian phase-type (PH) distribution. First, we show that different choices of parameters in our model lead to a range of tree balances as measured by Aldous’ $\beta$ statistic. In particular, we demonstrate that it is possible to find parameters that correspond well to empirical tree balance. Next, we provide a natural extension of the $\beta$ statistic to sets of trees. This extension produces less biased estimates of $\beta$ compared to using the median $\beta$ values from individual trees. Furthermore, we derive a likelihood expression for the probability of observing an edge-weighted tree under a model with speciation but no extinction. Finally, we illustrate the application of our model by performing both absolute and relative goodness-of-fit tests for two large empirical phylogenies (squamates and angiosperms) that compare models with Coxian PH distributed times to speciation with models that assume exponential or Weibull distributed waiting times. In our numerical analysis, we found that, in most cases, models assuming a Coxian PH distribution provided the best fit.

## Introduction

Understanding how biodiversity is maintained and changed throughout time has been of long-standing interest in evolutionary biology (Quental and Marshall 2010; Morlon 2014). Fossil records are commonly used to make inferences about changes through time in speciation and extinction rates (Simpson 1944; Stanley 1998; Morlon et al. 2011). However, most clades do not possess sufficiently complete fossil records to make such inferences (Ricklefs 2007; Quental and Marshall 2010). In contrast, dated molecular trees are increasingly available; nevertheless, these “reconstructed phylogenies” only give relationships between extant species (Nee et al. 1992, 1994a; Stadler 2013b). These reconstructed phylogenies can also be used to study how diversification processes change throughout time (Nee et al. 1994a), although some have argued that the use of reconstructed phylogenies needs to be accompanied with availability of fossil records (Quental and Marshall 2010; Morlon 2014; Hagen et al. 2018). However, reconstructed phylogenies remain useful to study diversification and diversity dynamics when accompanied by biologically well-justified constraints (Louca and Pennell 2020).

Several mathematical models have been proposed for studying macroevolutionary processes. These range from the constant-rate birth and death (crBD) model where speciation and extinction rates are assumed to be constant through time (Nee et al. 1994b), to models where speciation and extinction rates change according to species age (Hagen et al. 2015), to models where an evolving trait can affect speciation and extinction rates (Maddison et al. 2007; FitzJohn 2012). For models under the general birth–death process, in which speciation and extinction rates can vary over time, a recent paper by Louca and Pennell (2020) shows that many parameter choices are indistinguishable as they generate the same expected lineage-through-time (LTT) plot. Despite the problems identified by Louca and Pennell (2020), these fitted parameters still provide some insight into speciation and extinction rates or structure of relationships between species through time (Harvey and Pagel 1991; Stadler 2013b).

Given a choice of a model, various methods can be applied to use empirical (or simulated) data such as branch lengths from reconstructed trees to estimate the parameters of the model. For example, it is possible to derive an expression for the likelihood of observing these branch lengths and find the best-fitting parameters of the model using maximum-likelihood estimation (MLE) to make inference about the speciation and extinction rates (Morlon et al. 2011). In order to see which model fits empirical data best, we can assess models via the likelihood ratio test (LRT) or the Akaike’s Information Criterion (AIC) (Anderson and Burnham 2004) or via the comparison of their simulated LTT plot, which counts the number of species that existed at each given time in the past, with an empirical LTT plot (Morlon 2014). Then, given a model with best choice of parameters, we can assess whether it fits well to the empirical data by comparing tree balance or tree topology and branch length distributions from empirical and simulated trees generated from the model.

The balance of a phylogenetic tree describes the branching pattern of the tree, ranging from imbalanced shape where sister clades tend to be very different in sizes to balanced shape where the clades are of similar sizes. Tree balance is important for understanding macroevolutionary dynamics on a tree (Hagen et al. 2015) as it gives indication of heterogeneity of diversification rate across the tree without requiring information on branch lengths. Several statistics for assessing tree balance have been proposed in the literature. These include the Colless index (Colless 1982), the Sackin index (Sackin 1972) and Aldous’ $\beta$ (Aldous 1996)—Section 3.3 of Steel (2016) gives a detailed description of all three measures. In this paper, we focus exclusively on the $\beta$ statistic as, unlike the other two statistics, it is easily comparable between trees of different size. The $\beta$ statistic arises as a parameter of the Aldous’ $\beta$-splitting model; in this model $\beta$ is in the range $[-2,\infty )$ where values close to $-\,2$ mean that taxa are likely to split into unbalanced subsets and large values mean that splits are likely to be balanced. Many models in phylogenetics fail to resemble empirical datasets which often have $\beta$ value around $-\,1$ (Aldous 1996). For example, the simplest macroevolutionary model is the pure birth model, also known as the Yule–Harding (YH) model (Yule 1925), where each species is equally likely to speciate. It has been shown that trees under this model have the expected value $\beta =0$ (Aldous 1996; Hagen et al. 2015). In other words, the YH model predicts trees that are too balanced compared to empirical data (Aldous 1996, 2001). Likewise, models that include diversity-dependent (Etienne et al. 2012) and time-dependent speciation and extinction have been shown to produce the same expected tree balance as the YH model (Lambert and Stadler 2013). These models fall under a general class of species-speciation-exchangeable models as described in Stadler (2013b). This suggests that this class of models is not adequate to explain the macroevolutionary dynamics that has produced empirical trees.

Another statistic that has been widely used to compare empirical trees with macro-evolutionary models is the $\gamma$ statistic. The $\gamma$ statistic was introduced in Pybus and Harvey (2000) and unlike the tree balance statistics it makes use of the branch lengths. The statistic is designed to have a zero mean standard normal distribution under a pure birth model. Negative values of $\gamma$ mean that more diversification has occurred earlier in the tree than expected under a pure birth model, i.e., the edges nearer the root tend to be shorter relative to the other edges. Correspondingly, positive values of $\gamma$ mean that more diversification has occurred later in the tree and that edges nearer the root tend to be relatively longer. It has been shown that $\gamma$ values for empirical phylogenies tend to be below 0, which has sometimes been taken to indicate a slowdown in the diversification rate (Phillimore and Price 2008; Rabosky and Lovette 2008; Morlon et al. 2010).

**Fig. 1:**
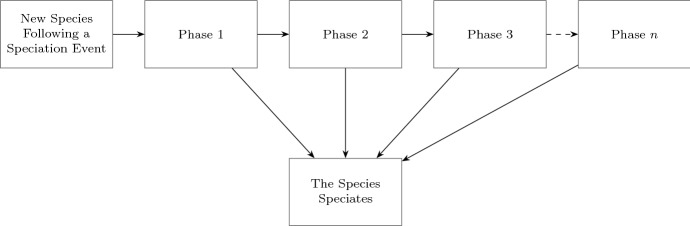
A new species passes through different phases during its ‘lifetime’ until the next speciation event. Each phase corresponds to a non-absorbing state in a CTMC and speciation corresponds to the single absorbing state. At the start, the species directly goes to phase 1 where it can either undergo speciation or move to the next phase with certain rates. The process can continue up to a finite number of *n* phases, each corresponding to a different rate of speciation

In this paper, we construct a stochastic model for generating species phylogenies in which we apply Coxian PH distributions (Neuts 1981; Marshall and McClean 2004) for times to speciation and times to extinction. PH distributions describe the time to absorption in a continuous-time Markov chain (CTMC) with a single absorbing state and a finite number of non-absorbing states. Biologically, this could be thought of as a species passing through different phases where it may be more or less likely to speciate depending on a current underlying phase (Fig. 1). While these phases need not represent any particular biological state, the PH distribution gives great flexibility to model different ways that rates of speciation may depend on a species’ age. Similarly, times to extinction can also be modeled using PH distributions. We show that different parameter choices for age-dependent speciation rates produce phylogenetic trees that can range from highly balanced to highly unbalanced. In particular, we find parameters that give similar tree balance statistics to empirical trees.

An additional contribution of the paper is that we develop a new approach for computing the $\beta$ statistic based on a set of trees rather than computing $\beta$ from a single tree. We suggest that this approach leads to more accurate estimates of the $\beta$ statistic compared to computing $\beta$ for single trees and then taking an average and that this is particularly true for trees with fewer extant species.

For a special case of our model, in which only speciation (and not extinction) occurs, we derive a likelihood expression for the probability of observing any edge-weighted tree. For two very large phylogenies—squamates (Zheng and Wiens 2016) and angiosperms (Zanne et al. 2014)—we perform model selection for different clades of both trees to compare our Coxian PH model for the speciation process to the exponential and Weibull distributions.

The rest of our paper is structured as follows. In the mathematical methods section we: (1) summarize the key properties of the PH distribution, (2) introduce some examples of Coxian PH distributions, (3) present our method for calculating the $\beta$ statistic for a set of trees, (4) and derive a likelihood expression based on our model for fitting empirical branch length data. The next section contains simulations that: (1) demonstrate the use of treeset $\beta$, (2) show that the model can produce trees with a wide range of tree shapes, (3) examine how well fitted models do in recovering the speciation process in scenarios with and without extinction. In the section on empirical data we apply our model to two large published phylogenies—squamates (Zheng and Wiens 2016) and angiosperms (Zanne et al. 2014). In summary, we find that Coxian PH distributions are a useful tool for studying macroevolutionary dynamics.

## Mathematical Methods

### PH Distribution and Relevant Properties

In this section, we introduce the PH distribution and some of its key properties.

#### Definition 1

(*Continuous PH distributions*) Let $\{X(t) : t \ge 0\}$ be a continuous time Markov chain defined on state space $S = \hat{S} \bigcup \{n+1\}$, where $\hat{S}=\{1,2,\ldots ,n\}$ is the set of non-absorbing states and $n+1$ is an absorbing state, initial distribution vector $\varvec{\alpha }=[\alpha _{i}]_{i \in \hat{S}}$, and generator matrix

1 $$\begin{aligned} \mathbf {Q^{*}} = [Q^{*}_{i,j}]_{i,j \in S} = \begin{bmatrix} \mathbf {Q} &{} \varvec{q}\\ \mathbf {0} &{}0 \end{bmatrix}, \end{aligned}$$

where $\mathbf {Q}=[Q_{i,j}]_{i,j\in \hat{S}}$ is a square matrix with dimension *n* that records the transition rates between non-absorbing states $i,j\in \hat{S}$, $\varvec{q}=[Q_{i,n+1}]_{i\in \hat{S}}$ is a column vector that records the transition rates from non-absorbing states $i \in \hat{S}$ to the absorbing state $n+1$, and $\mathbf {0}$ is the row vector with corresponding dimension. By the definition of generator matrix $\mathbf {Q}$, we have $Q_{i,i} < 0,\text {for all } i$, $Q_{i,j} \ge 0$ for $i \ne j$, and $\mathbf {Q}\mathbf {1}+\varvec{q}=\mathbf {0}$, where $\varvec{q}$ is the exit rate vector.

Let $Z=\text {inf}\left\{ t \ge 0 : X(t)=n+1\right\}$ be the random variable recording the time until absorption, then *Z* is said to be continuous PH distributed with parameters $\varvec{\alpha }$ and $\mathbf {Q}$, which we denote $Z \sim \text {PH} \left( \varvec{\alpha },\mathbf {Q}\right)$.

#### Theorem 1

(The cumulative distribution and density functions of continuous PH distribution) Suppose $Z \sim \mathrm{PH}\left( \varvec{\alpha },\mathbf {Q}\right)$, then the cumulative distribution and the probability density function of *Z* are given, respectively, by

2 $$\begin{aligned}&F_{Z}(z) = 1-\varvec{\alpha }e^{\mathbf {Q}z}\mathbf {1}, \end{aligned}$$

3 $$\begin{aligned}&f_{Z}(z) = \varvec{\alpha }e^{\mathbf {Q}z}\varvec{q}, \end{aligned}$$

and its mean and variance are given by

4 $$\begin{aligned}&E(Z)= -\varvec{\alpha }\mathbf {Q}^{-1}\mathbf {1}, \end{aligned}$$

5 $$\begin{aligned}&Var(Z)= 2\varvec{\alpha }\mathbf {Q}^{-2}\mathbf {1} -\left( \varvec{\alpha }\mathbf {Q}^{-1}\mathbf {1}\right) ^{2}. \end{aligned}$$

Proof of this theorem is originally given in Neuts (1975), and a clear exposition is given in Verbelen (2013). $\square$

#### Definition 2

(*Coxian PH distribution*) If $\varvec{\alpha }$ and $\mathbf {Q}$ are defined as

6 $$\begin{aligned} \varvec{\alpha }= & {} [1,0,\ldots ,0], \end{aligned}$$

7 $$\begin{aligned} \mathbf {Q}= & {} \begin{bmatrix} -\lambda _{1} &{} p_{1}\lambda _{1}&{} 0 &{} \dots &{} 0 &{} 0\\ 0 &{} -\lambda _{2} &{} p_{2}\lambda _{2} &{} \ddots &{} 0 &{} 0 \\ \vdots &{} \ddots &{} \ddots &{} \ddots &{} \ddots &{} \vdots \\ 0 &{} 0 &{} \ddots &{} -\lambda _{n-2} &{} p_{n-2}\lambda _{n-2} &{} 0\\ 0 &{} 0 &{} \dots &{} 0 &{} -\lambda _{n-1} &{} p_{n-1}\lambda _{n-1} \\ 0 &{} 0 &{} \dots &{} 0 &{} 0 &{} -\lambda _{n} \end{bmatrix}, \end{aligned}$$

where $0 < p_{i} \le 1$ and $\lambda _{1},\ldots ,\lambda _{n}>0$ for all $i = 1,2,\dots n-1$, then we say that the random variable $T \sim \mathrm{PH}\left( \varvec{\alpha },\mathbf {Q}\right)$ follows Coxian PH distribution.

Cumani (1982) showed that any acyclic PH (APH) distribution (including Coxian PH distributions), that is, a distribution with an upper triangular generator matrix (Asmussen et al. 1996), can be restructured to a canonical form such as shown above and thus only requires 2*n* parameters as opposed to $n^{2}+n$ parameters for a general PH distribution. This reduction in the number of parameters makes it computationally simpler to fit parameters (Thummler et al. 2006). Further, Cumani (1982) and Dehon and Latouche (1982) showed that for any APH distribution, there exists an equivalent representation as a Coxian PH distribution with $\lambda _{1}\le \lambda _{2} \le \cdots \le \lambda _{n}$.

To fit a PH distribution to data it is necessary to fix the number of non-absorbing states. Thummler et al. (2006) stated that it is difficult to fit general PH distributions if the number of non-absorbing states is larger than four, due to the increased computational cost and the dependence on the initial values. They also state that having a PH distribution of low order (less than four non-absorbing states) is not sufficient to get parameter values that correspond to small coefficients of variation (CV).

In Sects. 2.3 and 3.2 where we simulate data under different conditions, we focus solely on PH distributions with four non-absorbing states. In Sect. 4, where we fit models to empirical data, we explore a wider range of options for the number of non-absorbing states.

### Coxian-Based Macro-Evolutionary Model

Now, we develop a stochastic model for generating species phylogenies, in which we assume that the time spent by each newly formed lineage before the next speciation or extinction event is drawn from a Coxian PH distribution. Our model is a special case of the well-studied Bellman–Harris model which allows any distribution of waiting times to extinction or speciation (Bellman and Harris 1948). This model is discussed in Hagen and Stadler (2018) and they provide an *R* package (Hagen and Stadler 2018) that allows users to simulate trees under a general Bellman–Harris model. However, while it is possible to simulate trees under this very general class of models, it is not possible to fit parameters of a general Bellman–Harris distribution to empirical data. A novelty of our approach is that we are able derive a likelihood expression for the probability of observing a reconstructed phylogeny under our model in the case with no extinction and that we can therefore fit parameters.

In our model, we primarily focus on symmetric speciation. This means that after a speciation event two “child” species are created that are identical and of age 0. Thus, each branch length on a given tree can be thought of as an independent random variable drawn from the imposed Coxian PH distribution. We also consider asymmetric speciation in which the “parent” species is considered to continue and one new “child” species is created with age 0. Both symmetric and asymmetric speciation modes are supported by the *R* package *TreeSimGM* (Hagen and Stadler 2018).

We also construct two examples of the Coxian PH distribution as given in Definition 2. We parameterize the two examples so as to enforce either monotonically increasing or monotonically decreasing rates of absorption. In Example 1, the rate of speciation (or extinction) decreases as species get older, and in Example 2 the rate of speciation (or extinction) increases as species get older. We chose a parameterization with three free variables (*x*, *y* and *z*), as this gives flexibility to pick instances of each example with a given mean and variance, while at the same time reducing the number of free parameters for faster computational time (Okamura and Dohi 2016). Moreover, these two examples follow canonical form 3 of an APH distribution as stated in Okamura and Dohi (2016) (see also the derivation of the form by Cumani 1982). Note that there are different parameterizations that can be derived from the general Coxian PH distribution defined in Definition 2 which have either decreasing or increasing rate. However, these particular examples still provide some flexibility to choose different parameter values that give a wide range of coefficients of variation (CV) needed in Sect. 3.2.

#### Example 1

(*Coxian PH Distributed Model for Decreasing Rate*)

8 $$\begin{aligned} \mathbf {Q}=\begin{bmatrix} -z &{} (1-y)z &{} 0 &{} 0 &{} \\ 0 &{} -(1+x) &{} \left( 1-y^{2}\right) (1+x) &{} 0 \\ 0 &{} 0 &{} -\left( 1+x^{2}\right) &{} \left( 1-y^{3}\right) \left( 1+x^{2}\right) \\ 0&{} 0 &{} 0 &{} -x^{3} \end{bmatrix}, \varvec{q}=\begin{bmatrix} yz \\ y^{2}(1+x) \\ y^{3}\left( 1+x^{2}\right) \\ x^{3} \end{bmatrix}, \end{aligned}$$

where $0<x\le 1$, $0<y<1$, $z \ge 2$ and $\varvec{q}$ is the exit rate vector.

The restrictions on *x* and *y* imply that each entry of the exit rate vector $\varvec{q}$ is less than the preceding entry.

#### Example 2

(*Coxian PH Distributed Model for Increasing Rate*)

9 $$\begin{aligned}&\mathbf {Q}=\begin{bmatrix} -\left( 1+x^3\right) &{} \left( 1-y^4\right) \left( 1+x^3\right) &{} 0 &{} 0 &{} \\ 0 &{} -\left( 1+x^2\right) &{} \left( 1-y^{3}\right) \left( 1+x^2\right) &{} 0 \\ 0 &{} 0 &{} -(1+x) &{} \left( 1-y^{2}\right) (1+x) \\ 0&{} 0 &{} 0 &{} -z \end{bmatrix},\nonumber \\&\varvec{q}=\begin{bmatrix} y^{4}\left( 1+x^{3}\right) \\ y^{3}\left( 1+x^{2}\right) \\ y^{2}\left( 1+x\right) \\ z \end{bmatrix}, \end{aligned}$$

where $0<x\le 1$, $0<y<1$, $z \ge 2$ and $\varvec{q}$ is the exit rate vector.

Here, the restrictions on *x* and *y* imply that each entry of the exit rate vector $\varvec{q}$ is greater than the preceding entry.

From now on, we refer Examples 1 and 2 as $\mathrm{PH}_{\mathrm{Dec}}$ and $\mathrm{PH}_{\mathrm{Inc}}$, respectively. By standard theory of the PH distribution, the first and second moments of the Coxian PH distribution in $\mathrm{PH}_{\mathrm{Dec}}$ and $\mathrm{PH}_{\mathrm{Inc}}$ are given by

10 $$\begin{aligned} {\mathbb {E}}_{\mathrm{PH}}(X)= & {} \frac{1}{z}+(1-y)\left( \frac{1}{1+x} +\left( 1-y^2\right) \left( \frac{1}{1+x^2}+\frac{1-y^3}{x^3}\right) \right) , \nonumber \\ {\mathbb {E}}_{\mathrm{PH}}\left( X^{2}\right)= & {} \frac{2}{z^{2}}+\frac{2(1-y)}{1+x}\nonumber \\&\left( \frac{1}{z}+\frac{1}{1+x}\right) +\frac{2(1-y)\left( 1-y^2\right) }{1+x^2} \left( \frac{1}{z}+\frac{1}{1+x}+\frac{1}{1+x^2}\right) \nonumber \\&+\frac{2(1-y)\left( 1-y^2\right) \left( 1-y^3\right) }{x^3} \left( \frac{1}{z}+\frac{1}{1+x}+\frac{1}{1+x^2}+\frac{1}{x^3}\right) , \end{aligned}$$

and

11 $$\begin{aligned} {\mathbb {E}}_{\mathrm{PH}}(X)= & {} \frac{1}{1+x^{3}}+\left( 1-y^{4}\right) \left( \frac{1}{1+x^{2}}+\left( 1-y^3\right) \left( \frac{1}{1+x} +\frac{1-y^2}{z}\right) \right) ,\nonumber \\ {\mathbb {E}}_{\mathrm{PH}}\left( X^{2}\right)= & {} \frac{2}{\left( 1+x^3\right) ^2} +\frac{2\left( 1-y^4\right) }{1+x^2}\left( \frac{1}{1+x^3}+\frac{1}{1+x^2}\right) +\frac{2\left( 1-y^4\right) \left( 1-y^3\right) }{1+x}\nonumber \\&\left( \frac{1}{1+x^3}+\frac{1}{1+x^2}+\frac{1}{1+x}\right) +\frac{2\left( 1-y^4\right) \left( 1-y^3\right) \left( 1-y^2\right) }{z}\nonumber \\&\left( \frac{1}{1+x^3}+\frac{1}{1+x^2}+\frac{1}{1+x}+\frac{1}{z}\right) ,\nonumber \\ \end{aligned}$$

respectively. The derivations of Eqs. 10 and 11 are shown in “Appendix.”

### Computing $\beta$ for a Set of Trees

We propose a new approach for estimating the tree-balance statistic $\beta$ from a set of rooted trees $\{T_1,\ldots , T_M\}$, which can be either empirical trees or simulated trees under some model of interest. For each subtree with four or more tips in each tree in $\{T_1,\ldots , T_M\}$ we compute the probability $q_{n}(i,\beta )$ of observing *i* tips on the left out of the *n* tips of that subtree. This is done using Eq. 4 from Aldous (1996),

12 $$\begin{aligned} q_{n}(i,\beta ) = \frac{1}{a_{n}(\beta )}\frac{\varGamma (\beta +i+1) \varGamma (\beta +n-i+1)}{\varGamma (i+1)\varGamma (n-i+1)},1 \le i \le n-1, \end{aligned}$$

where $a_{n}(\beta )$ is the normalizing constant. We note that subtrees of size 2 or 3 are not of interest as there is only one possible division of the tips. In the case where the tree size is too large, the above expression is not numerically tractable, so we use the following approximation instead (which is also used in the *apTreeShape* package (Bortolussi et al. 2006)), given by

13 $$\begin{aligned} q_{n}(i,\beta ) = \frac{1}{\hat{a}_{n}(\beta )} \left( \frac{i}{n}\right) ^{\beta }\left( 1-\frac{i}{n}\right) ^{\beta }, \end{aligned}$$

where $\hat{a}_{n}(\beta )$ is the normalizing constant. (Justification for the approximation in Eq. 13 is given in “Appendix.”)

We then use numerical optimization to find the value of $\beta$ in the range $[-2, 10]$ which maximizes the product of all the $q_{n}(i,\beta )$ values. This is the maximum likelihood estimate of $\beta$ for the set of trees. Our custom *R* script, based on *maxlik.betasplit* function from the *apTreeShape* package (Bortolussi et al. 2006) to estimate $\beta$ from sets of trees, is available as a Supplementary Material on Dryad (https://doi.org/10.5061/dryad.w9ghx3fpk).

### Fitting PH Distributions to Branch Length Data

In this section, we propose a method for finding parameters of a PH distribution using branch length data from a phylogenetic tree. We assume that the time until a speciation event on a branch follows a PH distribution and that there is no extinction. We write the likelihood expression using parameters from the PH distribution to calculate the probability of observing a tree with a given number of extant species.

**Fig. 2:**
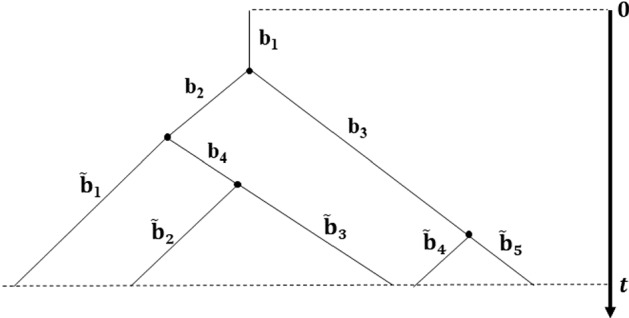
Phylogenetic tree with five extant tips evolving under a symmetric speciation mode. Branch lengths are independent and drawn from the same PH distribution $\mathrm{PH}\left( \varvec{\alpha },\mathbf {Q}\right)$

Assuming that a tree evolves under a symmetric speciation mode, and that times to speciation events are drawn from a PH distribution, we can treat each branch length on the tree as independently drawn from the same PH distribution. We illustrate this in Fig. 2, in which the lengths of internal branches and pendant branches are denoted by $\{b_{1},b_{2},b_{3},b_{4}\}$ and $\{\tilde{b}_{1}, \tilde{b}_{2},\tilde{b}_{3},\tilde{b}_{4},\tilde{b}_{5}\}$, respectively.

In general, we denote the lengths of internal and pendant branches by $b_i$, for $i=1,\ldots ,k$, and $\tilde{b}_j$, for $j=1,\ldots ,\ell$, where the total number of internal branches and pendant branches is denoted by *k* and $\ell$, respectively. Here, because we consider the root branch, we note that $k=\ell -1$. Both internal and pendant branches follow a PH distribution with parameter $\varvec{\alpha }$ and rate matrix $\mathbf {Q}$, that is, $b_i,\tilde{b}_j \sim \mathrm{PH}\left( \varvec{\alpha },\mathbf {Q}\right)$. It follows from the properties of the PH distribution (Neuts 1981), that the likelihood of observing an internal branch of length $b_i$ is the probability density of the distribution along the branch given by $\varvec{\alpha } e^{\mathbf {Q}b_{i}}\varvec{q}$ and the likelihood of observing a pendant branch of length $\tilde{b}_j$ is the probability that the branch has survived until time t (i.e., one minus the cumulative probability of the distribution) given by $\varvec{\alpha }e^{\mathbf {Q} \tilde{b}_j}\varvec{1}$, where $\varvec{1}$ is a column vector of ones. Therefore, by independence of the branch lengths, the likelihood of observing tree *T* can be written as,

14 $$\begin{aligned} {\mathcal {L}}\left( T\,\vert \,\varvec{\alpha },\mathbf {Q}\right) =\prod _{i=1}^{k}\left( \varvec{\alpha }e^{\mathbf {Q}b_{i}}\varvec{q}\right) \times \prod _{j=1}^{\ell }\left( \varvec{\alpha }e^{\mathbf {Q}\tilde{b}_{j}}\varvec{1}\right) , \end{aligned}$$

with $\varvec{\alpha } = [1,0,\ldots ,0]$, since we apply Coxian PH distribution. Note that if we consider all the possible permutations on the tips of the tree, then the likelihood becomes,

15 $$\begin{aligned} {\mathcal {L}}\left( T\,\vert \,\varvec{\alpha },\mathbf {Q}\right) = (\ell -1)! \times \prod _{i=1}^{k}\left( \varvec{\alpha } e^{\mathbf {Q}b_{i}}\varvec{q}\right) \times \prod _{j=1}^{\ell } \left( \varvec{\alpha }e^{\mathbf {Q}\tilde{b}_{j}}\varvec{1}\right) . \end{aligned}$$

Given the branch lengths of a *single tree* *T*, we perform numerical optimization to find parameter values that maximize the likelihood equation given in Eq. 14. In the case of the general Coxian PH model this amounts to finding the best values of $p_{i}$’s and $\lambda _{i}$’s as in Definition 2, for $\mathrm{PH}_{\mathrm{Dec}}$ and $\mathrm{PH}_{\mathrm{Inc}}$ it means finding the best values of *x*, *y*, and *z*.

Alternatively, given the branch lengths of a *tree set* $\{T_1,\ldots , T_M\}$, we apply maximum likelihood estimation to maximize the product

16 $$\begin{aligned} {\mathcal {L}}\left( \{T_1,\ldots , T_M\}\,\vert \,\varvec{\alpha },\mathbf {Q}\right) ={\mathcal {L}}\left( T_{1}\,\vert \,\varvec{\alpha },\mathbf {Q}\right) \times \cdots \times {\mathcal {L}}\left( T_{M}\,\vert \,\varvec{\alpha },\mathbf {Q}\right) , \end{aligned}$$

where we assume trees are independent and apply Eq. 14 to compute the likelihood of observing the individual trees $T_1,\ldots , T_M$.

To optimize parameters for the exponential and Weibull distribution, we derive an equivalent expression to Eq. 14 for both distributions. The likelihood expression for the exponential distribution is given by

17 $$\begin{aligned} {\mathcal {L}}\left( T\,\vert \,\lambda \right) = \prod _{i=1}^{k} \lambda \exp ^{-\lambda b_{i}}\times \prod _{j=1}^{\ell }\exp ^{-\lambda \tilde{b}_{j}}, \end{aligned}$$

and for the Weibull distribution

18 $$\begin{aligned} {\mathcal {L}}\left( T\,\vert \,\psi ,\phi \right) =\prod _{i=1}^{k}\frac{\psi }{\phi }\left( \frac{b_i}{\phi }\right) ^{\psi -1} \exp ^{-(b_{i}/\phi )^\psi }\times \prod _{j=1}^{\ell } \exp ^{-\left( \tilde{b}_{j}/\phi \right) ^\psi }, \end{aligned}$$

where $\psi$ and $\phi$ are scale and shape parameters, respectively.

Then, we apply maximum likelihood estimation to search for $\lambda > 0$ that maximizes Eq. 16. Similarly, we search for $\psi > 0$ and $\phi > 0$ parameters that maximize Eq. 16.

Finally, we consider a birth-and-death process (BDP) with constant birth rate $\lambda$ and constant death rate $\mu$. The likelihood expression for the reconstructed tree under such BDP is given in Eq. 20 of Nee et al. (1994b), it is a conditional probability conditioning on the survival of both original branches descending from the root.

**Fig. 3:**
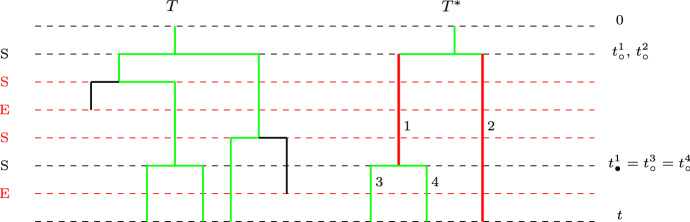
Original tree *T* and the reconstructed tree $T^*$, where S (in black) denotes observed speciation events and E (in red) denotes unobserved extinction events. Speciation events S (in red) followed by extinction events E (in red) on the original tree *T*, are not observed on the branches (highlighted in red) of the reconstructed tree $T^*$. The internal branch 1 on $T^*$ was born at time $t_{\circ }^{1}$ and the next *observed* speciation event on that branch (on $T^*$) was at time $t_{\bullet }^{1}$. The external branch 2, 3,  and 4 on $T^*$ was born at time $t_{\circ }^{2},t_{\circ }^{3},$ and $t_{\circ }^{4}$, respectively, and no speciation events are observed on that branch (on $T^*$) (Color figure online)

Note that the likelihood for the reconstructed tree under any process that includes extinction events needs to consider the possibility that speciation events that end with extinction may occur on internal or external branches and so are not observed on the reconstructed tree (see Fig. 3).

Below, we present our alternative likelihood formula for the reconstructed tree under a BDP. This formula provides new physical interpretations given by Eqs. 19–23, in the context of the dynamics of the process driving the evolution of the phylogenetic tree in time.

Assume that *t* is the age of the tree with 0 is the time at the start of the root branch and let $x_i$ be the elapsed time from the end of the internal branch *i* until the end of tree *T*. That is, if internal branch *i* is born at time $t_{\circ }^{i}$ and gives birth at time $t_{\bullet }^{i}$ to another branch, then $x_i=t-t_{\bullet }^{i}$ and its length is $b_i=t_{\bullet }^{i}-t_{\circ }^{i}$. For the external branch *j* descending from the internal branch *i*, we have its branch length given by $\tilde{b}_j=t-t_{\bullet }^{i}=x_i$.

Then, the likelihood of observing a reconstructed species tree $T^*$ is given by

19 $$\begin{aligned} {\mathcal {L}}(T^* \ |\ \lambda , \mu )= (\ell -1)! \prod _{i=1}^k G_{x_i,t}(b_i) \lambda \prod _{j=1}^{\ell }D^{(1)}_{t}(\tilde{b}_j), \end{aligned}$$

where $G_{x_i,t}(b_i)$ is the probability of observing reconstructed internal branch *i*, and $D^{(1)}_{t}(\tilde{b}_j)$ is the probability of observing reconstructed external branch *j*, where $G_{x,t}(z)$ is the solution of

20 $$\begin{aligned} G_{x,t}(z)= & {} e^{-(\lambda +\mu )z}+\int _{u=0}^{z}{e^{-(\lambda +\mu )(z-u)} \lambda \left( 2G_{x,t}(u)E(u+x)\right) \mathrm{d}u}, \end{aligned}$$

21 $$\begin{aligned} \frac{\mathrm{d}G_{x,t}(z)}{\mathrm{d}z}= & {} -(\lambda +\mu )G_{x,t}(z)+2\lambda G_{x,t}(z)E(z+x), \end{aligned}$$

and $D^{(1)}_{t}(z)$ is the solution of

22 $$\begin{aligned} D^{(1)}_{t}(z)= & {} e^{-(\lambda +\mu )z}+\int _{u=0}^{z}{e^{-(\lambda +\mu )u} \lambda \left( 2D^{(1)}_{t}(z-u)E(z-u)\right) \mathrm{d}u}, \end{aligned}$$

23 $$\begin{aligned} \frac{\mathrm{d}D^{(1)}_{t}(z)}{\mathrm{d}z}= & {} -(\lambda +\mu )D^{(1)}_{t}(z) + 2\lambda E(z)D^{(1)}_{t}(z) , \end{aligned}$$

where by Kendall (1948)

24 $$\begin{aligned} E(z)= \frac{\mu -\mu e^{(\mu -\lambda )z}}{\lambda -\mu e^{(\mu -\lambda )z}} \end{aligned}$$

is the probability that a branch born at time zero becomes extinct by time *z*. Solving the above equations gives

25 $$\begin{aligned} G_{x,t}(z)= & {} \left( \frac{\lambda -\mu e^{(\mu -\lambda )x}}{\lambda - \mu e^{(\mu -\lambda )(z+x)}}\right) ^{2}e^{(\mu -\lambda )z}, \end{aligned}$$

26 $$\begin{aligned} D^{(1)}_{t}(z)= & {} \left( \frac{(\lambda -\mu )e^{\mu z}}{\lambda -\mu e^{(\mu -\lambda )z}}\right) ^{2}e^{-(\lambda +\mu )z}. \end{aligned}$$

The derivation of the differential equations for $$D^{(1)}_{t}(z)$$ and $$ G_{x,t}(z)$$ along with their solutions and some intuition are shown in “Appendices 6.4 and 6.5.”

Next, we apply our likelihood expression in Eq. 19 to the reconstructed tree $T^*$ in Fig. 3 (ignoring the age of the root) to see that

27 $$\begin{aligned} {\mathcal {L}}_{\mathrm{Nee}}(T^* \ |\ \lambda , \mu ) = \frac{{\mathcal {L}}(T^* \ |\ \lambda , \mu )}{\left( 1-E(x_{2})\right) ^{2}}, \end{aligned}$$

where ${\mathcal {L}}_{\mathrm{Nee}}(T^* \ |\ \lambda , \mu )$ is the likelihood expression given in Eq. 20 in Nee et al. (1994b), and $x_{2}$ is the elapsed time from the starting time of the two original branches descending from the root until the end of the tree $T^*$, as defined in Nee et al. (1994b). This relationship is as expected, since ${\mathcal {L}}_{\mathrm{Nee}}(T^* \ |\ \lambda , \mu )$ is a conditional probability of observing the tree $T^*$ given that both original branches have survived until the end of the tree.

## Simulations

### Comparing Treeset $\beta$ to the Standard $\beta$

To compare $\beta$ values estimated from individual trees to those estimated for a set of trees, we performed the following simulation. We simulated sets of 1000 trees using *TreeSimGM* package (Hagen and Stadler 2018), where each set of trees had the same number of extant tips $n\in \{10,20,30,\ldots ,200\}$ and their times to speciation were drawn from PH distribution with rate matrix

28 $$\begin{aligned} \mathbf {Q}=\begin{bmatrix} -2 &{} 1 &{} 0 &{} 0 &{} \\ 0 &{} -1.1 &{} 1 &{} 0 &{} \\ 0 &{} 0 &{} -1.01 &{} 1 \\ 0 &{} 0 &{} 0 &{} -0.001 \end{bmatrix}, \quad \varvec{q} =\begin{bmatrix} 1 \\ 0.1 \\ 0.01\\ 0.001\\ \end{bmatrix}. \end{aligned}$$

We note that the structure of the exit rate vector $\varvec{q}$ implies that the probability of getting absorbed from later states is less likely than from earlier states. We then repeated the above procedure for sets of trees evolving under the YH model. The YH case is interesting because it is representative of a wider class of models that are known to have $E(\beta )=0$ (Aldous 2001).

For each set of trees, we computed individual estimates of $\beta$ for each tree as well as a $\beta$ estimate for the entire tree set. We also computed $95\%$ confidence intervals for the estimated $\beta$ values, denoted $\hat{\beta }$, from each tree set. In order to get the lower and upper bound for the confidence intervals, we performed a numerical search over 500 equidistant points between $\hat{\beta }-5\times SE\left( \hat{\beta }\right)$ and $\hat{\beta }$ to find the point that corresponds to the lower bound and 500 equidistant points between $\hat{\beta }$ and $\hat{\beta }+5\times SE\left( \hat{\beta }\right)$ to find the point that corresponds to the upper bound. The lower and upper bounds were chosen such that their likelihood is equal to the likelihood of the MLE minus a half of the chi-square value with 1 degree of freedom; this gives a 95% confidence interval (Pawitan 2001). The standard error for $\hat{\beta }$, $SE\left( \hat{\beta }\right)$, was evaluated using

29 $$\begin{aligned} SE\left( \hat{\beta }\right) = \frac{1}{\sqrt{I\left( \hat{\beta }\right) }}, \end{aligned}$$

where $I\left( \hat{\beta }\right)$ is the Fisher information of $\hat{\beta }$.

**Fig. 4:**
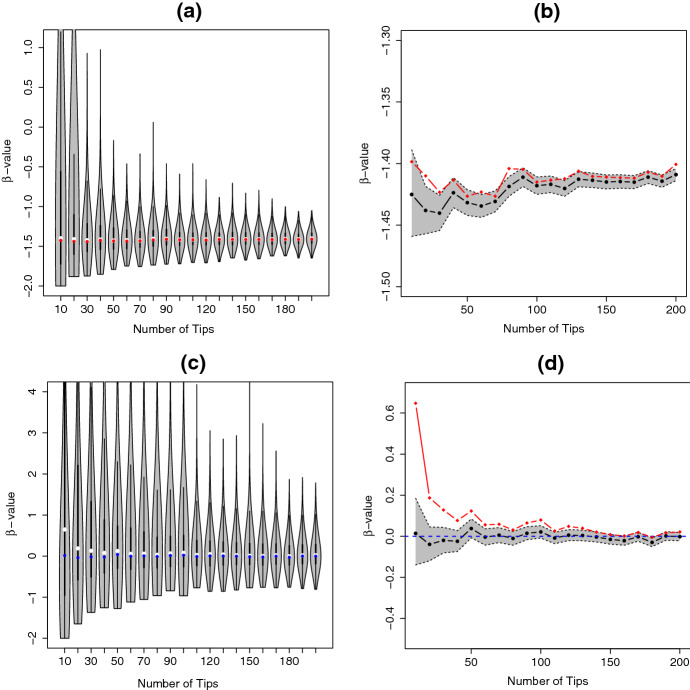
Estimates of $\beta$ for individual trees with $n \in \{10,20,30,\ldots ,200\}$ tips (**a**–**d**). Estimates of $\beta$ from treesets are indicated by red dots (**a**) and blue dots (**c**). Trees are simulated according to either Coxian PH distribution for times to speciation events (**a**) or the YH process (**c**). The area of $95\%$ confidence interval of $\beta$ values from treesets following Coxian PH distribution and the YH process are plotted in (**b**), (**d**), respectively. The black lines represent the treeset $\beta$ values, and the gray area represents the confidence interval for each treeset $\beta$ value. The red lines represent the median $\beta$ values from individual trees. The blue-dashed line represents the theoretical $\beta$ value for the YH trees $\left( \beta =0\right)$ (Color figure online)

The results are summarized in Fig. 4. For both of the generating processes, the distribution of $\beta$ values is right-skewed (Fig. 4a, c) and the median value for individual trees is higher than the value estimated using the entire tree set particularly for trees with fewer tips (Fig. 4b, d). For the trees generated under the YH process, when estimating the value of $\beta$ for trees with fewer extant tips we obtained $\beta \approx 0$ when applying the method based on treesets, but median $\beta >0$ for estimates based on individual trees (Fig. 4c, d). We conclude that the method based on treesets is more accurate for the Yule process, as evidenced by the $95\%$ confidence interval in Fig. 4d. The $\beta$ values estimated from different sets of trees concentrate around $\beta = 0$ in agreement with the theoretical value for trees evolving under the YH model. We think that the upwards bias in estimation of $\beta$ arises because, for trees with fewer tips, it not unlikely to get a tree that is maximally balanced (or close to it) and in this case the maximum likelihood procedure for fitting $\beta$ prefers to make $\beta$ as large as possible.

### Coxian-PH Models can Generate a Range of Tree Shapes

In Hagen et al. (2015), the authors found that using a Weibull distribution for age dependent speciation had an effect on tree balance (as measured by the $\beta$ statistic), whereas using a Weibull distribution for extinction had an effect on diversification (as measured by the $\gamma$ statistic). To test if using PH distributions gives similar results, we simulated trees using the two examples $\mathrm{PH}_{\mathrm{Dec}}$ and $\mathrm{PH}_{\mathrm{Inc}}$. We did not see obvious changes in the $\beta$ and $\gamma$ statistics under different parameter values using $\mathrm{PH}_{\mathrm{Inc}}$, so we only report results for $\mathrm{PH}_{\mathrm{Dec}}$. The simulation procedure was a follows:

- As an example, we set $z=10$ and mean waiting time to both speciation and extinction ${\mathbb {E}}_{\mathrm{PH}}(X) = 2$. The choice of ${\mathbb {E}}_{\mathrm{PH}}(X)$ scales the branch lengths of generated phylogenies, but results will be invariant to this choice of the mean since we only consider tree balance and relative branch lengths. Likewise, the *z* parameter is chosen arbitrarily as long as it is larger than or equal to 2 in order to preserve a decreasing rate as described in $\mathrm{PH}_{\mathrm{Dec}}$.
- We then selected 4 pairs of parameters $0<x\le 1$ and $0<y<1$ to give a wide range of coefficients of variation (CV). We found choices of *x* and *y* where $\text {CV} = \frac{\sigma }{\mu } \in \{30.08,13.50,5.56,1.49\}$. These 4 pairs of *x* and *y* are as follows: $(x,y) \in \{(0.1,0.93),(0.17,0.88), (0.3,0.78), (0.68,0.45)\}$. We also note that fixing either *x* or *y* parameters gives less flexibility in choosing (*y*, *z*) or (*x*, *z*) pairs corresponding to a wide range of CV.
- Using the *TreeSimGM* package (Hagen and Stadler 2018) in *R*, we generated 300 trees with 100 extant tips in which times to speciation followed a PH distribution with parameters *x*, *y* and *z*, while times to extinction followed an exponential distribution with rate $\lambda =0.25$. The main goal in choosing trees of size 100 was to have trees that were large enough for $\beta$ to be accurately estimated for individual trees, but small enough to have reasonable running time. We repeated this procedure for both symmetric and asymmetric speciation modes. Then we repeated everything again but using an exponential distribution for the times to speciation (with $\lambda =20$) and the PH distributions described above for the times to extinction.
- We measured the effect of different parameter choices above on tree balance using the $\beta$ statistic. We computed the $\beta$ statistic both for individual trees, using the *apTreeshape* package (Bortolussi et al. 2006), and for sets of trees based on our new approach. We also measured the effect on relative branch lengths as measured by the $\gamma$ statistic (Pybus and Harvey 2000), which we computed using the *APE* package (Paradis et al. 2004).

**Fig. 5:**
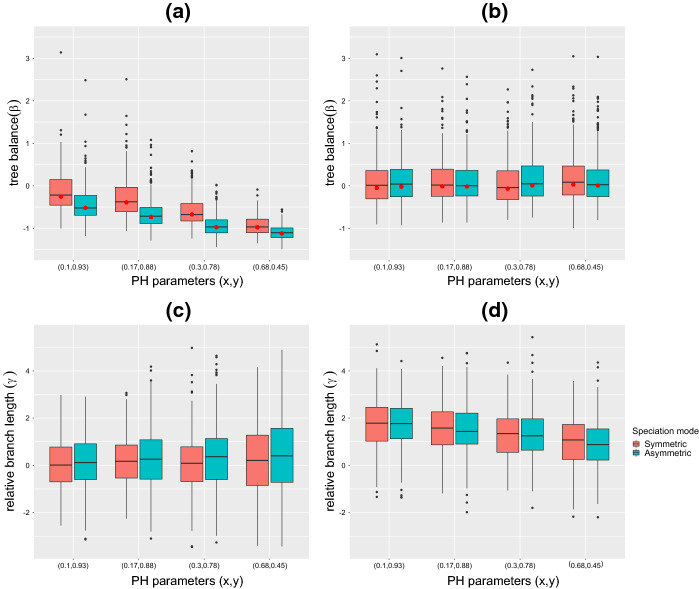
Effect of speciation and extinction processes on tree balance as measured by the $\beta$ statistic, and on relative branch lengths as measured by the $\gamma$ statistic. For each pair of parameters (*x*, *y*) in $\mathrm{PH}_{\mathrm{Dec}}$ used to generate either times to speciation (in **a**, **c**) or times to extinction (in **b**, **d**), we simulated 300 trees with 100 extant tips. In **a**, **c** the parameters of the times to speciation are $(x,y) \in \{(0.1,0.93), (0.17,0.88), (0.3,0.78), (0.68,0.45)\}$ and mean speciation time is ${\mathbb {E}}_{\mathrm{PH}}(X)=2$, while times to extinction are drawn from exponential distribution with rate $\lambda =0.25$. In **b**, **d** times to speciation are drawn from exponential distribution with rate $\lambda =20$, while the parameters of the times to extinctions are $(x,y) \in \{(0.1,0.93),(0.17,0.88),(0.3,0.78),(0.68,0.45)\}$ and mean extinction time is ${\mathbb {E}}_{\mathrm{PH}}(X)=2$. The red dots show the $\beta$ statistic for sets of trees (Color figure online)

The results are presented in Fig. 5. Tree balance is affected by varying the parameters for times to speciation (Fig. 5a), in particular, there are choices of model parameters that match the tree-shape statistics of empirical phylogenies ($\beta ~= -1$). Tree balance is not significantly affected by the parameters for times to extinction (Fig. 5b). In contrast to the behavior of $\beta$, relative branch lengths, as measured by the $\gamma$ statistic are not affected by the parameters for times to speciation (Fig. 5c), while they are affected by the parameters for times to extinction (Fig. 5d). We did not observe a significant difference in our results between the symmetric and asymmetric speciation modes. These results are congruent with what was found in Hagen et al. (2015).

### Fitting Coxian-PH Distributions to Branch Length Data

In this section, we test if the maximum likelihood approach outlined in Sect. 2.4 is able to fit the speciation process well in cases where: (a) there is no extinction, and (b) the generating model includes extinction. As an example to illustrate the bias introduced by not considering the extinction process in the likelihood function in Eq. 16, we simulated trees using the $\mathrm{PH}_{\mathrm{Dec}}$ distribution with known parameter values, for the speciation process and an exponential distribution for the extinction process with rate $\lambda \in \{0, 0.1, 0.4\}$, and then fitted the parameters of the $\mathrm{PH}_{\mathrm{Dec}}$ distribution to the generated branch length data. In total, we generated 50 trees with 50 extant tips each, using *TreeSimGM* package (Hagen and Stadler 2018), which produced 4900 branches.

Using Eq. 14–16, we found the parameters *x*, *y*, and *z* that maximized the likelihood of observing the given set of branch lengths. The optimization was carried out using the built-in *R* function, *optim*, with the “*L-BFGS-B*” method (Byrd et al. 1995) and multiple starting points for *x*, *y*, *z*, followed by local optimization using the “Nelder-Mead” method (Nelder and Mead 1965).

**Fig. 6:**
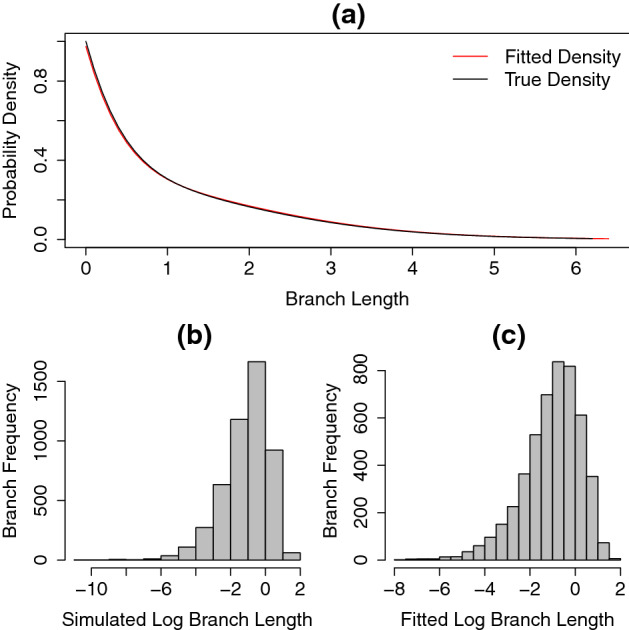
True density function (black line in **a**) and the fitted density function (red line in **a**) from $\mathrm{PH}_{\mathrm{Dec}}$ distribution. The values of *x*-axis on panel **a** show the branch lengths from both fitted and simulated trees. The histograms of the simulated and fitted branch length distributions, shown in log scale, are displayed in (**b**), (**c**), respectively. Data were simulated with no extinction (Color figure online)

**Fig. 7:**
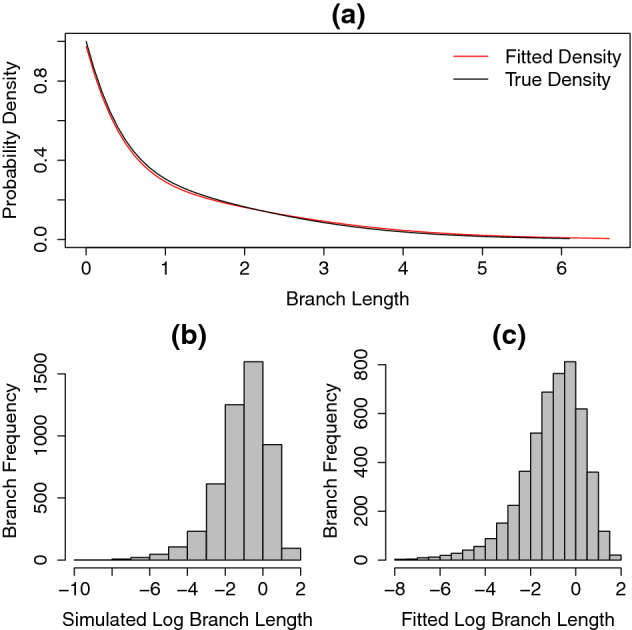
True density function (black line in **a**) and the fitted density function (red line in **a**) from $\mathrm{PH}_{\mathrm{Dec}}$ distribution. The values of *x*-axis on panel **a** show the branch lengths from both fitted and simulated trees. The histograms of the simulated and fitted branch length distributions, shown in log scale, are displayed in (**b**), (**c**), respectively. Here, extinction events follow an exponential distribution with rate $\lambda =0.1$ (Color figure online)

**Fig. 8:**
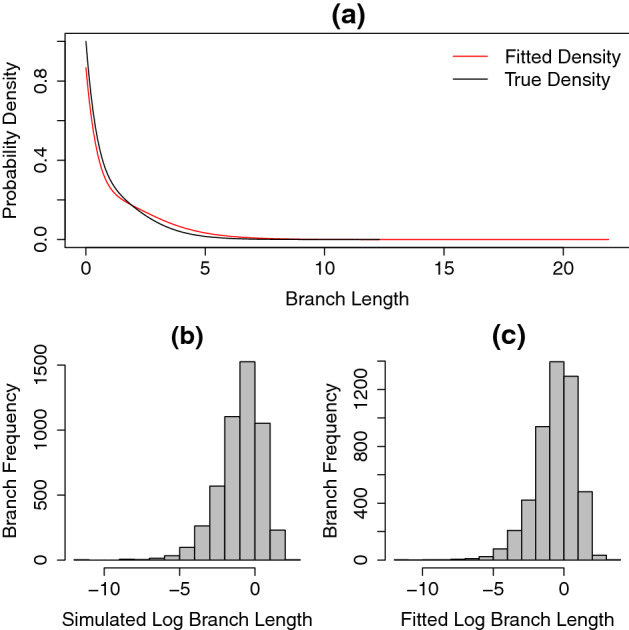
True density function (black line in **a**) and the fitted density function (red line in **a**) from $\mathrm{PH}_{\mathrm{Dec}}$ distribution. The values of *x*-axis on panel **a** show the branch lengths from both fitted and simulated trees. The histograms of the simulated and fitted branch length distributions, shown in log scale, are displayed in (**b**), (**c**), respectively. Here, extinction events follow an exponential distribution with rate $\lambda =0.4$ (Color figure online)

To compare the fitted distribution to the generating distribution we plotted the density of the fitted distribution and the known distribution used to simulate the data. Additionally, using the fitted parameters *x*, *y* and *z*, we generated trees with the same number of tips as in the simulated data, and compared their distribution of branch lengths with that of the simulated trees. Note that we cannot simply compare the branch length histogram from trees generated under the known distribution with its fitted frequency density plot since the generated trees are truncated at some time *t* (the tree’s age). Therefore, to compare distributions of branch lengths we used the two sample Kolmogorov-Smirnov (KS) test of the null hypothesis that both simulated and fitted log branch lengths come from the same distribution (using the built-in *ks.test* function in $\textit{R}$). The results of this analysis are shown in Figs. 6, 7, 8 and Table 1.

In the scenario without extinction (Fig. 6) the fitting process was able to recover the parameters since the generated trees do not assume extinction, the KS statistic found no significant difference in the log branch lengths produced by the true generating model and the fitted $\mathrm{PH}_{\mathrm{Dec}}$ model (Table 1). In the scenarios that included extinction, the fitting process was not able to correctly recover the true generating model (Figs. 7, 8). The bias in estimating the speciation process becomes more apparent as we increase the extinction rate (Fig. 8).

**Table 1:** KS tests for hypothesis testing that both fitted and simulated log branch lengths in Fig. 6, 7 and 8 come from the same distribution

Extinction rate $\lambda$	KS Statistic	*p* value
0	0.025	0.101
0.1	0.034	0.007
0.4	0.115	$\ll$ 0.001

## Empirical Data

In this section, we apply the techniques developed in Sect. 2.4 to two large empirical phylogenies (Zheng and Wiens 2016; Zanne et al. 2014). In order to view these phylogenies and to extract clades of interest, we used Dendroscope 3 software (Huson and Scornavacca 2012). For each dataset, we compared nine models. These included models where the speciation process followed a PH distribution: the general Coxian distribution (Definition 2) with 3, 4, 5, and 6 non-absorbing states, and the two examples $\mathrm{PH}_{\mathrm{Dec}}$ and $\mathrm{PH}_{\mathrm{Inc}}$ developed in Sect. 2.2, one model where the speciation process follows an exponential distribution, one where it follows a Weibull distribution, and one where we fit to the constant rate birth–death model (crBD) using the likelihood formula of observing a tree conditioned on survival in Eq. 20 in Nee et al. (1994b) or using the likelihood in Eq. 27. We note that our likelihood formula as in Eq. 14 does not consider permutation on the tips of tree, so it differs from the likelihood from the crBD model by $(N-1)!$ where *N* denotes the number of tips on tree.

Our general approach for model comparison was to use the Akaike Information Criterion (AIC) (Akaike 1998) which is essentially the log likelihood penalized according to the number of parameters used in the model. We followed the approach suggested in Anderson and Burnham (2004) which is that models with an AIC difference ($\varDelta$AIC) of less than two are essentially as good as the best model, and models with $\varDelta$AIC less than 6 should not be discounted.

In addition to assessing relative goodness-of-fit via the AIC, and bearing in mind that all of our models are likely to be wrong given that they ignore extinction, we also assessed absolute goodness-of-fit using the KS statistic to compare fitted branch length densities to empirical branch length densities.

Lastly we show the hazard rate function for speciation from the best-fitting model for each clade. We were interested to see how different these would be to the constant hazard rate assumed by most macroevolutionary models or the monotonically decreasing hazard rate given by a Weibull distribution.

### Squamate Phylogeny

We fit the models under consideration to the branch lengths from the squamate phylogeny in Zheng and Wiens (2016). We also examined three major clades of the tree separately, namely the *gekkota* clade (1318 branches), the *iguania* clade (1936 branches), and the *anguimorpha* clade (200 branches), to see if there are any notable differences.

The model comparison results are summarized in Table 2. The general Coxian model is strongly preferred for the overall tree and for all the clades being studied. In particular, the general Coxian model with three non-absorbing states fits best, but the model with four non-absorbing states is essentially indistinguishable. Additionally, fitting to the $\mathrm{PH}_{\mathrm{Inc}}$ example model is significantly worse than other distributions. Moreover, fitting to the crBD model returns zero extinction rate for all the cases and returns the same parameter values for speciation process, comparable to the model that follows exponential speciation rate without extinction.

**Fig. 9:**
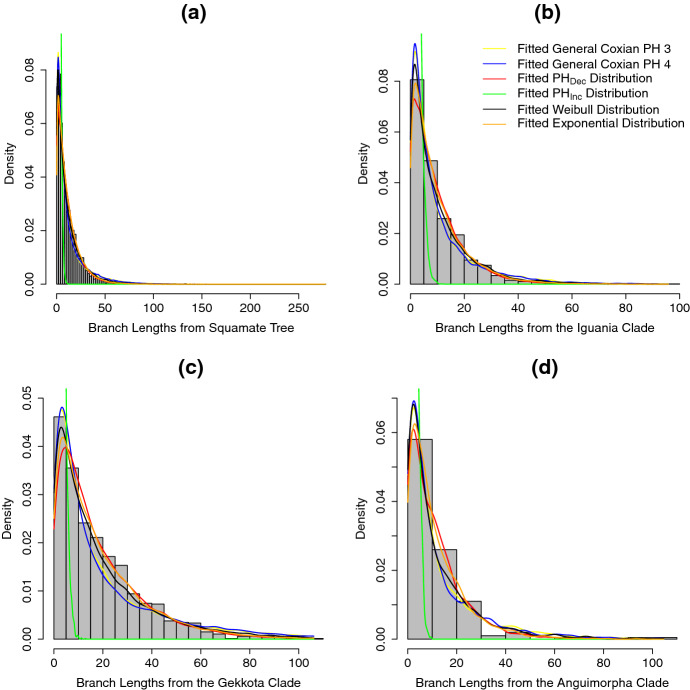
Histograms of empirical branch length density from the whole squamate tree (**a**), the *iguania* clade (**b**), the *gekkota* clade (**c**), and the *anguimorpha* clade (**d**) with the fitted branch length densities from the six distributions mentioned above. The yellow and blue lines are the fitted densities using the general Coxian PH distribution defined in Definition 2 with 3 and 4 non-absorbing states, respectively, the red line is the fitted density using the Coxian $\mathrm{PH}_{\mathrm{Dec}}$ example, the green line is the fitted density using the Coxian $\mathrm{PH}_{\mathrm{Inc}}$ example, and black and orange lines are the fitted density using Weibull and exponential distributions, respectively. The fitted densities for the general Coxian PH distribution with 5 and 6 non-absorbing states are not included because in most cases the distribution with 3 and 4 non-absorbing states fit better, while the fitted density from the crBD model is not included because it is identical to the fitted density using exponential distribution (see Table 2) (Color figure online)

The absolute goodness-of-fit of different models is assessed in Fig. 9. Visually both general Coxian PH distribution with three and four non-absorbing states give fairly similar densities. These two appear to fit better compared to the other distributions (in agreement with the AIC results in Table 2). Both of these distributions seem to capture the tail behavior fairly well, but do a poorer job of matching the density for shorter branch lengths. The lack of fit to the reconstructed squamate tree and to most clades is supported by the KS tests which show a significant difference between the empirical branch lengths and branch lengths of 10 simulated trees from each best-fitting distribution (Table 3). We use the phytools package (Revell 2012) to simulate trees under the crBD model. Given that earlier results (Hagen et al. (2015) and Fig. 5d) show that the extinction process affects relative branch lengths, we hypothesize that this result could be due to ignoring extinction events in the models. Interestingly, all the distributions, except for $\mathrm{PH}_{\mathrm{Inc}}$, show a good fit between the empirical branch lengths of the *anguimorpha* clade and branch lengths of 10 simulated trees from each of these best-fitting distributions (Table 3). We note that this result could be due to the clade having a relatively small number of extant tips (101 tips); therefore, there is a lack of power to distinguish between models. Alternatively, extinction may occur at a lower rate in this clade compared to the other clades.

**Table 2:** Model selection is based on the likelihood of observing the branch lengths given the specified model for times to speciation and no extinction (as per Sect. 2.4)

Model	# branches	# parameters	LogL	AIC	$\varDelta$AIC
(a)					
General Coxian PH 3	8336	5	$-$16727.93	33465.86	0
General Coxian PH 4		7	$-$16727.02	33468.04	2.18
General Coxian PH 5		9	$-$16726.87	33471.74	5.88
General Coxian PH 6		11	$-$16727.22	33476.44	10.58
$\mathrm{PH}_{\mathrm{Dec}}$		3	$-$17793.08	35592.15	2126.29
$\mathrm{PH}_{\mathrm{Inc}}$		3	$-$68410.67	136827.34	103361.50
Exponential distribution		1	$-$17322.14	34646.29	1180.43
Weibull distribution		2	$-$17024.64	34053.27	587.41
Constant rate birth–death		2	$-$17322.14	34648.29	1182.43
(b)					
General Coxian PH 3	1318	5	$-$2958.58	5927.16	0
General Coxian PH 4		7	$-$2958.08	5930.15	2.99
General Coxian PH 5		9	$-$2958.48	5934.96	7.80
General Coxian PH 6		11	$-$2958.51	5939.02	11.86
$\mathrm{PH}_{\mathrm{Dec}}$		3	$-$3092.96	6191.91	264.75
$\mathrm{PH}_{\mathrm{Inc}}$		3	$-$18783.21	37572.42	31645.25
Exponential distribution		1	$-$3048.34	6098.69	171.53
Weibull distribution		2	$-$3006.61	6017.21	90.05
Constant rate birth–death		2	$-$3048.34	6100.69	173.53
(c)					
General Coxian PH 3	1936	5	$-$3775.66	7561.33	0
General Coxian PH 4		7	$-$3774.20	7562.39	1.07
General Coxian PH 5		9	$-$3773.78	7565.55	4.23
General Coxian PH 6		11	$-$3773.75	7569.49	8.17
$\mathrm{PH}_{\mathrm{Dec}}$		3	$-$3963.97	7933.93	372.61
$\mathrm{PH}_{\mathrm{Inc}}$		3	$-$12852.02	25710.05	18148.72
Exponential distribution		1	$-$3860.30	7722.61	161.28
Weibull distribution		2	$-$3827.23	7658.46	97.14
Constant rate birth–death		2	$-$3860.30	7724.61	163.28
(d)					
General Coxian PH 3	200	5	$-$398.14	806.28	0
General Coxian PH 4		7	$-$398.11	810.22	3.94
General Coxian PH 5		9	$-$398.12	814.25	7.96
General Coxian PH 6		11	$-$398.08	818.17	11.88
$\mathrm{PH}_{\mathrm{Dec}}$		3	$-$417.02	840.05	33.76
$\mathrm{PH}_{\mathrm{Inc}}$		3	$-$1607.49	3220.98	2414.69
Exponential distribution		1	$-$410.18	822.37	16.08
Weibull distribution		2	$-$402.24	808.47	2.18
Constant rate birth–death		2	$-$410.18	824.37	18.08

The constant rate birth–death (crBD) model is the only model that includes extinction in this comparison. The numbers (3, 4, 5, 6) in the row labels for the general Coxian PH indicate the number of non-absorbing states. For the crBD model from Nee et al. (1994b), we adjust the log likelihood by subtracting $\log ((\ell -1)!)$ where $\ell$ is the number of tips on tree. We select the model that has the lowest AIC value as the base model and compute $\varDelta$AIC$=\text {AIC}_{\mathrm{other} \ \mathrm{model}}-\text {AIC}_{\mathrm{best}\ \mathrm{model}}$. We use branch lengths from (a) the whole reconstructed squamate tree; and from different clades from the tree, namely (b) the *gekkota* clade, (c) the *iguania* clade, and (d) the *anguimorpha* clade

**Table 3:** KS tests for hypothesis testing that empirical branch length data of the reconstructed squamate tree and its following clades come from these fitted distributions

Distribution	Squamate	Iguania	Gekkota	Anguimorpha
	KS statistic	KS statistic	KS statistic	KS statistic
Constant rate birth–death	0.028	0.038	0.037	0.071
General Coxian PH 3	0.063	0.059	0.079	0.085
General Coxian PH 4	0.059	0.063	0.075	0.091
General Coxian PH 5	0.059	0.062	0.076	0.085
General Coxian PH 6	0.057	0.059	0.072	0.075
$\mathrm{PH}_{\mathrm{Dec}}$	0.062	0.059	0.061	0.069
$\mathrm{PH}_{\mathrm{Inc}}$	0.576	0.550	0.716	0.572
Weibull	0.069	0.064	0.071	0.081
Exponential	0.030	0.033	0.041	0.046

For the reconstructed squamate tree, the *iguania*, and the *gekkota* clades, the resulting *p* values from these KS statistics are all significant ($p< 0.05$), indicating that branch lengths drawn from these fitted distributions are significantly different than the empirical branch lengths. However, in the case of the *anguimorpha* clade, the *p* value are not significant ($p> 0.05$) in most distributions, except for $\mathrm{PH}_{\mathrm{Inc}}$. This indicates that branch lengths drawn from these fitted distributions are not statistically different compared to the empirical branch lengths

**Fig. 10:**
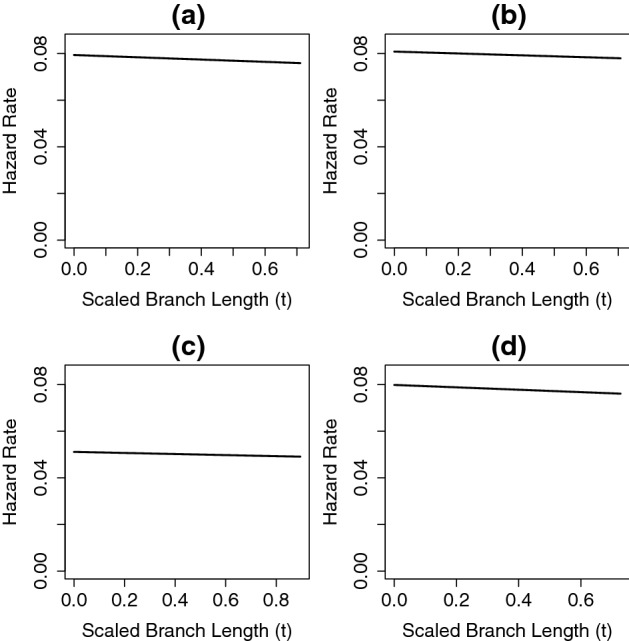
Hazard rate functions for speciation show the change in the instantaneous probability of speciation as species age, as determined using the best-fitting general Coxian PH distribution with four non-absorbing states for the whole squamate tree (**a**), the *iguania* clade (**b**), the *gekkota* clade (**c**), and the *anguimorpha* clade (**d**). For each tree, branches are scaled by dividing each branch length leading to speciation event with height of the tree

The hazard rate functions for speciation from the best-fitting general Coxian PH distribution with four non-absorbing states for each the overall squamate phylogeny and the three major clades are shown in Fig. 10. For the overall tree and for each clade, the instantaneous rate of speciation seems to show a slight decline (almost constant) as species get older.

### Angiosperm Phylogeny

To see how each model performs on an even larger tree, we also fit branch lengths from four different clades of the angiosperm phylogeny of (Zanne et al. 2014). The four different clades we use are: the *monocotyledoneae* clade (14,118 branches), the *magnoliidae* clade (2092 branches), the *superrosidae* clade (11,323 branches), and the *superasteridae* clade (20,016 branches).

**Fig. 11:**
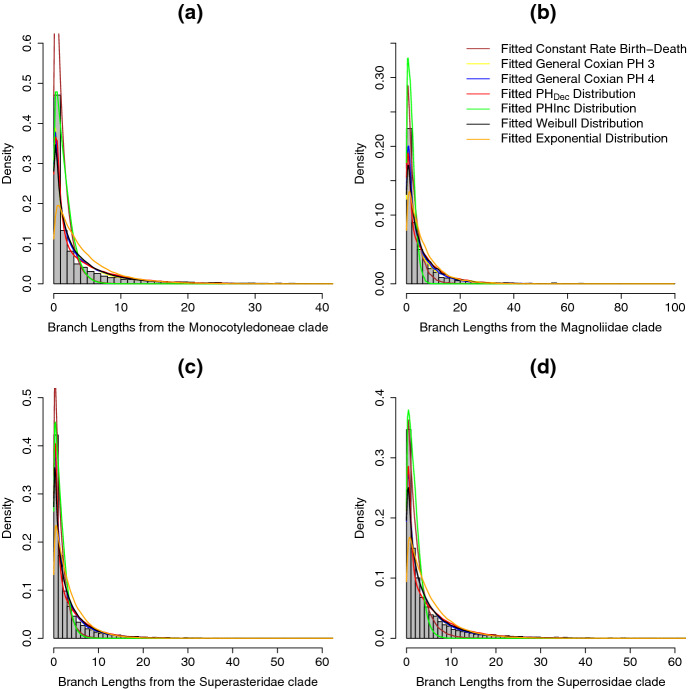
Histograms of empirical branch length density from the *monocotyledoneae* clade (**a**), the *magnoliidae* (**b**), the *superasteridae* clade (**c**) and the *superrosidae* clade (**d**) with the fitted branch length densities from the six distributions mentioned earlier. The yellow and blue lines are the fitted densities using the general Coxian PH distribution defined in Definition 2 with 3 and 4 non-absorbing states, respectively, the red line is the fitted density using the Coxian $\mathrm{PH}_{\mathrm{Dec}}$ example, the green line is the fitted density using the Coxian $\mathrm{PH}_{\mathrm{Inc}}$ example, and black and orange lines are the fitted density using Weibull and exponential distributions, respectively. The fitted densities for the general Coxian PH distribution with 5 and 6 non-absorbing states are not included because in most cases having the distribution with less number of non-absorbing states (e.g., four non-absorbing states) fit better (see Table 4) (Color figure online)

The model comparison results are summarized in Table 4. The general Coxian model are very strongly preferred over all the other models for all of the individual clades. Additionally, fitting to the model that follows $\mathrm{PH}_{\mathrm{Inc}}$ example is significantly worse than other distributions. Moreover, unlike the results in Table 2, the general Coxian model with four non-absorbing states fit best in this case. Interestingly, fitting to the crBD model to this set of empirical data returns non-zero extinction rate for all of the individual clades and it fits better compared to the model following an exponential speciation rate without extinction. The absolute goodness-of-fit of different models is assessed in Fig. 11. Visually, both general Coxian PH distributions with three and four non-absorbing states give fairly similar densities. These two appear to fit better compared to the other distributions (in agreement with the AIC results in Table 4). Both of these distributions seem to capture the tail behavior fairly well, but do a poorer job of matching the density for shorter branch lengths. The lack of fit is supported by the KS tests which show a significant difference between the empirical branch lengths and branch lengths of 10 simulated trees from each best-fitting distribution (Table 5). Again, we hypothesize that this result could be due to ignoring extinction events in the model. Here, as with the squamate data, we observe that the density of the fitted distribution of $\mathrm{PH}_{\mathrm{Inc}}$, which imposes increasing speciation rates as species age, does not follow the shape of the empirical histograms for any of the clades (Fig. 11).

**Table 4:** Model selection is based on the likelihood of observing the branch lengths given the specified model for times to speciation and no extinction (as per Sect. 2.4)

Model	# branches	# parameters	LogL	AIC	$\varDelta$AIC
(a)					
General Coxian PH 3	14118	5	$-$18498.30	37006.59	112.74
General Coxian PH 4		7	$-$18439.92	36893.85	0
General Coxian PH 5		9	$-$18439.56	36897.13	3.28
General Coxian PH 6		11	$-$18439.46	36900.92	7.07
$\mathrm{PH}_{\mathrm{Dec}}$		3	$-$18788.38	37582.76	688.91
$\mathrm{PH}_{\mathrm{Inc}}$		3	$-$45591.17	91188.34	54294.49
Exponential distribution		1	$-$22149.68	44301.37	7407.52
Weibull distribution		2	$-$18633.08	37270.16	376.31
Constant rate birth–death		2	$-$19979.50	39962.99	3069.14
(b)					
General Coxian PH 3	2092	5	$-$3369.34	6748.68	40.07
General Coxian PH 4		7	$-$3347.30	6708.60	0
General Coxian PH 5		9	$-$3346.96	6711.93	3.33
General Coxian PH 6		11	$-$3346.95	6715.89	7.29
$\mathrm{PH}_{\mathrm{Dec}}$		3	$-$3476.51	6959.01	250.41
$\mathrm{PH}_{\mathrm{Inc}}$		3	$-$8926.00	17857.99	11149.39
Exponential distribution		1	$-$3633.44	7268.87	560.27
Weibull distribution		2	$-$3395.17	6794.34	85.73
Constant rate birth–death		2	$-$3493.25	6990.51	281.90
(c)					
General Coxian PH 3	20016	5	$-$29808.68	59627.35	117.45
General Coxian PH 4		7	$-$29747.95	59509.90	0
General Coxian PH 5		9	$-$29747.57	59513.15	3.25
General Coxian PH 6		11	$-$29747.56	59517.11	7.21
$\mathrm{PH}_{\mathrm{Dec}}$		3	$-$30533.85	61073.71	1563.81
$\mathrm{PH}_{\mathrm{Inc}}$		3	$-$59551.17	119108.33	59598.44
Exponential distribution		1	$-$33668.54	67339.07	7829.17
Weibull distribution		2	$-$30064.19	60132.39	622.49
Constant rate birth–death		2	$-$31765.43	63534.87	4024.97
(d)					
General Coxian PH 3	11323	5	$-$29977.30	59964.60	0
General Coxian PH 4		7	$-$29977.32	59968.64	4.04
General Coxian PH 5		9	$-$29977.33	59972.66	8.06
General Coxian PH 6		11	$-$29977.35	59976.71	12.11
$\mathrm{PH}_{\mathrm{Dec}}$		3	$-$30717.66	61441.32	1476.72
$\mathrm{PH}_{\mathrm{Inc}}$		3	$-$72613.88	145233.76	85269.16
Exponential distribution		1	$-$33136.25	66274.49	6309.89
Weibull distribution		2	$-$30183.84	60371.68	407.08
Constant rate birth–death		2	$-$31791.90	63587.81	3623.21

The constant rate birth–death (crBD) model is the only model that includes extinction in this comparison. The numbers (3, 4, 5, 6) in the row labels for the general Coxian PH indicate the number of non-absorbing states. For the crBD model from Nee et al. (1994b), we adjust the log likelihood by subtracting $\log ((\ell -1)!)$ where $\ell$ is the number of tips on tree. We select the model that has the lowest AIC value as the base model and compute $\varDelta$AIC$=\text {AIC}_{\mathrm{other model}}-\text {AIC}_{\mathrm{best model}}$. We use branch lengths from different clades of the angiosperm phylogeny, namely (a) the *monocotyledoneae* clade, (b) *magnoliidae* clade, (c) *superasteridae* clade and (d) the *superrosidae* clade

**Fig. 12:**
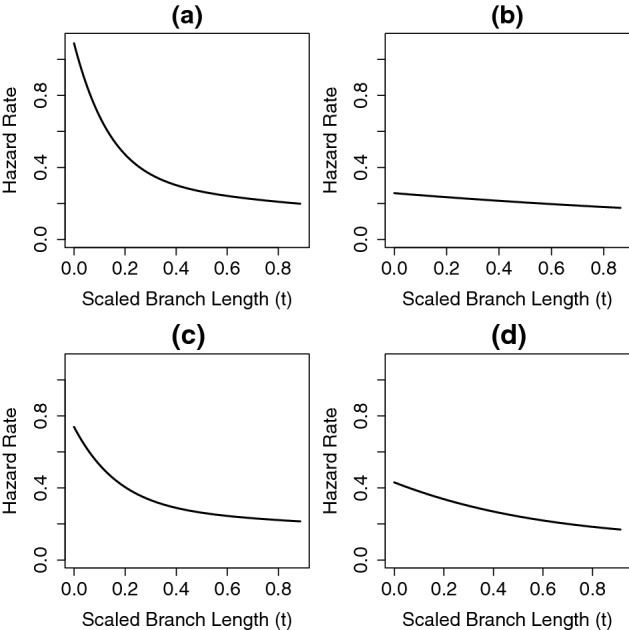
Hazard rate functions for speciation show the change in the instantaneous probability of speciation as species age, as determined using the best-fitting general Coxian PH distribution with four non-absorbing states for the *monocotyledoneae* clade (**a**), the *magnoliidae* clade (**b**), the *superasteridae* clade (**c**), and *superrosidae* clade (**d**)

The hazard rate functions for speciation from the best-fitting general Coxian PH distribution with four non-absorbing states for the four major clades of the angiosperm phylogeny are shown in Fig. 12. The instantaneous rate of speciation declines in each case and the rate of decline appears to be different in major clades of the angiosperm tree.

## Discussion and Conclusion

Our macroevolutionary model for phylogenetic trees where times to speciation or extinction events are drawn from a Coxian PH distribution can produce phylogenetic trees with a range of tree shapes. The model provides a good fit to empirical data compared to exponential and Weibull distributions. The idea of applying PH distributions is motivated by the following two properties. First, it is well known that PH distributions are dense in the field of all positive-valued distributions (Asmussen et al. 1996), and thus, they are very flexible when fitting to empirical distributions. In particular, it implies that waiting times to either speciation or extinction events that follow any positive real-value distributions, such as exponential and Weibull, are well approximated using PH distribution with some given structure. Second, evolution of species trees or a species tree can be modeled as a forward-in-time process which follows an acyclic PH distribution. It is also known in the literature that any acyclic PH distribution can be represented as a Coxian PH distribution (Cumani 1982; Asmussen et al. 1996). Using a Coxian distribution is particularly useful here because its structure allows for the process to reach the absorbing state from any of the non-absorbing states, as described in Definition 2. This implies, using a general Coxian PH distribution, we can create an example where either speciation or extinction rates decrease or increase over time, by only changing parameter values inside the rate matrix $\mathbf {Q}$, such as ones in $\mathrm{PH}_{\mathrm{Dec}}$ and $\mathrm{PH}_{\mathrm{inc}}$. However, we recommend using the general Coxian PH distribution when used to fit to empirical data.

**Table 5:** KS tests for hypothesis testing that empirical branch length data of the following clades from the reconstructed angiosperm come from these fitted distributions

Distribution	Monocotyledoneae	Magnoliidae	Superasteridae	Superrosidae
	KS statistic	KS statistic	KS statistic	KS statistic
Constant rate birth–death	0.232	0.169	0.179	0.178
General Coxian PH 3	0.080	0.057	0.048	0.044
General Coxian PH 4	0.082	0.039	0.042	0.043
General Coxian PH 5	0.078	0.047	0.042	0.044
General Coxian PH 6	0.076	0.047	0.043	0.040
$\mathrm{PH}_{\mathrm{Dec}}$	0.073	0.072	0.045	0.055
$\mathrm{PH}_{\mathrm{Inc}}$	0.217	0.290	0.188	0.264
Weibull	0.100	0.080	0.066	0.061
Exponential	0.261	0.173	0.183	0.178

The resulting *p* values from these KS statistics are all significant $(p < 0.05)$, indicating that branch lengths drawn from these fitted distributions are significantly different than the empirical branch lengths

We have demonstrated that trees generated under our model can have a range of different levels of tree balance as measured by the $\beta$ statistic (Fig. 5). Thus, it is possible to fit parameters of our model to empirical tree shapes. The ability to get tree shapes that vary from the uniform distribution on ranked tree shapes (URT) in our model is expected based on the work of Lambert and Stadler (2013). A model with Coxian PH distributed times to speciation and exponentially distributed times to extinction is in class 4 of the scheme given in Lambert and Stadler (2013), in which the speciation process depends on a non-heritable trait (in this case species age).

In our simulations, we found that tree balance is mainly controlled by the speciation process and is largely invariant to the extinction process. In contrast to the behavior of $\beta$, the relative branch lengths, as measured by the $\gamma$ statistic, are to a large extent controlled by the extinction process, but relatively invariant to the speciation process. Interestingly, unlike the $\beta$ statistic where we found model parameters that gave values around $-1$, we did not find any model parameters that led to negative values of $\gamma$. We also found that using symmetric or asymmetric speciation modes did not have much effect on tree balance. These findings agree with the results in Hagen et al. (2015) in which speciation and extinction processes were modeled using Weibull distribution.

We proposed a method of computing the $\beta$ statistic based on sets of trees. We have demonstrated that computing the $\beta$ statistic based on individual trees can be upwardly biased, particularly for trees with smaller numbers of taxa. For trees generated by a YH process, computing the $\beta$ statistic based on sets of trees gives a more accurate result (Fig. 4). This approach of computing a $\beta$ value for a set of trees is useful in the context of simulated tree data, but beyond simulation studies, there may be other contexts where it is useful to estimate $\beta$ for a set of trees. For example, when studying bio-geographic patterns researchers may have multiple species trees for the same set of geographic regions. It would also be possible to compute a single $\beta$ value for a set of gene trees.

We derived a likelihood expression for the probability of observing any reconstructed tree (Eqs. 14–16) that has evolved with PH distributed times to speciation (and no extinction); we applied it to both simulated and empirical data by applying the maximum likelihood method. We note that fitting parameters based on branch lengths taken from trees that include extinction, produces some bias in estimation of the speciation process (Fig. 7). The bias becomes more apparent with increasing rates of extinction (Fig. 8). In future work, we aim to generalize Eq. 14 to include extinction. Such an extension can potentially be done in a similar manner as the derivation for the likelihood under a BDP process as described in Eq. 19. Once we derive a generalized likelihood function, we will compare its performance with likelihood functions that consider both speciation and extinction events, such as in Rabosky (2006).

In Sect. 2.4, we have also given a different approach for deriving the likelihood expression of observing a tree evolving under a constant rate birth–death process. This expression in Eq. 19 provides new physical interpretations in the context of the process driving the evolution of phylogenetic tree, and it also has a nice relationship with the formula in Nee et al. (1994b) as described in Eq. 27. In terms of fitting the model to empirical data, we note that the likelihood must be conditioned on the survival of the original two branches descending from the root of the tree as seen in Eq. 27. This agrees with what Stadler (2013a) stated in her paper.

Finally, we have fitted the parameters of our model to the empirical data consisting of branch lengths from various clades in the squamate and angiosperm reconstructed phylogenies (Zheng and Wiens 2016; Zanne et al. 2014). In both cases, we found that the extra flexibility permitted by the Coxian PH distribution was favored by the AIC over the simpler Weibull and Exponential models. Interestingly, in both cases, the model using the Coxian PH distribution without extinction process still fits better than the constant rate birth–death model from Nee et al. (1994b) that includes extinction. Moreover, in one example, fitting using the Coxian PH distribution with three non-absorbing states is preferable, but fitting using the distribution with four non-absorbing states is mostly preferred. Meanwhile, fitting to the same distribution with more than four non-absorbing states was always less favorable in the examples we looked at while also adding more computational time.

In the squamate phylogeny (Zheng and Wiens 2016), all the clades we examined (*iguania*, *gekkota*, *anguimorpha*) showed rates of speciation that declined slightly as species got older (Fig. 10). The whole squamate phylogeny also showed slight declining rates of speciation (almost constant rate). On the other hand, two clades (*monocotyledoneae*, *superasteridae*) from angiosperm phylogeny (Zanne et al. 2014) considered in this study showed apparent declining rates of speciation as species got older (Fig. 12a, c), while the other clades in the phylogeny (*magnoliidae*, *superrosidae*) only showed rates of speciation that decreased slightly (Fig. 12b, d). We caution against reading too much into these results as the model does not include extinction or account for incomplete sampling.

In summary, we have demonstrated that our macroevolutionary model with Coxian PH distribution, provides a better fit to empirical phylogenies, when compared to models with other distributions, including exponential and Weibull (Tables 2, 4). We conclude that it is necessary to use distributions with sufficient complexity, such as Coxian PH distributions, to provide a better fit to empirical phylogenies.

## Figures and Tables

**Fig. 13:**
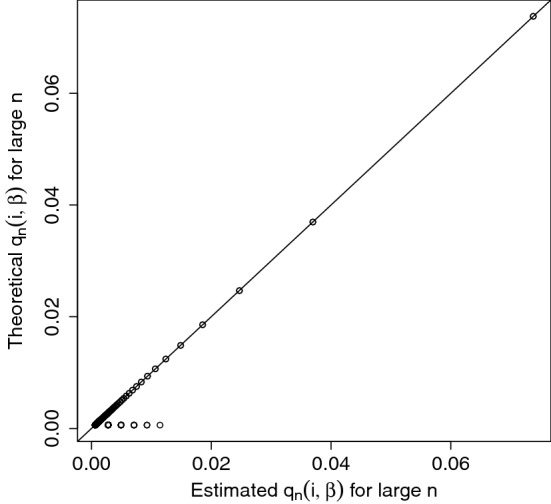
Comparison of the probability $q_{n}(i,\beta )$ defined in Eqs. 35 and 30 for $n = 500$ and $\beta = -1$. The *x*-axis represents the probability defined in Eq. 35 while the *y*-axis represents the probability defined in Eq. 30

## Data Availability

The datasets and all the relevant code, including functions for fitting empirical data to a phase-type model and for computing treeset $\beta$ values, are available in the DRYAD repository, https://doi.org/10.5061/dryad.w9ghx3fpk

## Appendix

### Equivalence of Formulas for $q_{n}(i,\beta )$

There are two different formulas for computing the probability of observing *i* tips on the left given *n* extant tips on a tree, $q_{n}(i,\beta )$. The first expression includes a product of gamma functions with a normalizing constant, $a_{n}(\beta )$, as seen in Eq. 4 from Aldous (1996), while the second expression includes a product of beta functions with a normalizing constant, $\hat{a_{n}}(\beta )$, as seen in the *maxlik.betasplit* command from *apTreeShape* package (https://github.com/bcm-uga/apTreeshape/blob/master/R/maxlik.betasplit.R). Here, we show that both expressions are equivalent by showing that both normalizing constants are related.

Recall from Aldous (1996), we have

30 $$\begin{aligned} q_{n}(i,\beta )=\frac{1}{a_{n}(\beta )}\frac{\varGamma (\beta +i+1) \varGamma (\beta +n-i+1)}{\varGamma (i+1)\varGamma (n-i+1)}, 1\le i \le n-1, \end{aligned}$$

where $a_{n}(\beta )$ is a normalizing constant and $\varGamma (x)$ is the gamma function.

Recall from the *maxlik.betasplit* command, we have

31 $$\begin{aligned} \hat{q}_{n}(i,\beta ) = \frac{1}{\hat{a_{n}}(\beta )} \frac{B(\beta +i+1,\beta +n-i+1)}{B(i+1,n-i+1)},1\le i \le n-1, \end{aligned}$$

where $\hat{a_{n}}(\beta )$ is a normalizing constant and *B*(*x*, *y*) is beta function.

#### Proof

Using the relation between gamma and beta functions where $B(x,y)=\frac{\varGamma (x)\varGamma (y)}{\varGamma (x+y)}$, we can write Eq. 31 as,

32 $$\begin{aligned} \hat{q}_{n}(i,\beta )&= \frac{1}{\hat{a_{n}}(\beta )} \frac{\frac{\varGamma (\beta +i+1)\varGamma (\beta +n-i+1)}{\varGamma (2\beta +n+2)}}{\frac{\varGamma (i+1)\varGamma (n-i+1)}{\varGamma (n+2)}} \end{aligned}$$

33 $$\begin{aligned}&= \frac{\varGamma (n+2)}{\hat{a_{n}}(\beta )\varGamma (2\beta +n+2)} \frac{\varGamma (\beta +i+1)\varGamma (\beta +n-i+1)}{\varGamma (i+1)\varGamma (n-i+1)} \end{aligned}$$

34 $$\begin{aligned}&= \frac{\varGamma (n+2)}{\hat{a_{n}}(\beta ) \varGamma (2\beta +n+2)}a_{n}(\beta )q_{n}(i,\beta ). \end{aligned}$$

Hence, $\hat{q}_{n}(i,\beta ) = q_{n}(i,\beta )$ if and only if $\frac{1}{\hat{a_{n}}(\beta )}=\frac{\varGamma (n+2)}{a_{n}(\beta )\varGamma (2\beta +n+2)}$. That is, $\hat{a}_{n}(\beta )=\frac{a_{n}(\beta ) \varGamma (2\beta +n+2)}{\varGamma (n+2)}$. $\square$

### Equivalent Formula of $q_{n}(i,\beta )$ for Large *n* and *i*

Here, we show the work to approximate Eqs. 30 and 31 for large *n* and *i*, where *n* is the number of extant tips on the tree and *i* is the number of left tips on the tree. We use this approximation due to computational limitation of evaluating gamma function for large number. The formula also appears in the *maxlik.betasplit* from the *TreeSimGM* package (Hagen and Stadler 2018).

#### Lemma 1

Given large *n* and *i*, Eqs. 30 and 31 can be approximated using the following formula,

35 $$\begin{aligned} \hat{q}_{n}(i,\beta ) = \frac{1}{\hat{a}_{n}(\beta )} \left( \frac{i}{n}\right) ^{\beta }\left( 1-\frac{i}{n}\right) ^{\beta }, \end{aligned}$$

where $\hat{a}_{n}(\beta )$ is the normalizing constant for $\hat{q}_{n}(i,\beta )$.

#### Proof

Recall the Stirling’s approximation for gamma function is given by

36 $$\begin{aligned} \varGamma (z) \approx \sqrt{\frac{2\pi }{z}}\left( \frac{z}{e}\right) ^{z}. \end{aligned}$$

Then, we claim that

#### Lemma 2

37 $$\begin{aligned} \frac{\varGamma (x+\beta +1)}{\varGamma (x+\alpha +1)} \approx x^{\beta -\alpha } \text { for large }x. \end{aligned}$$

#### Proof

By Stirling’s approximation with $z=x+\beta$ and $z=x+\alpha$, we have

38 $$\begin{aligned} \frac{\varGamma (x+\beta +1)}{\varGamma (x+\alpha +1)}&= \frac{(x+\beta )\varGamma (x+\beta )}{(x+\alpha ) \varGamma (x+\alpha )}, \text {since}\ x+\beta \text {and}\ x+\alpha \in Z \end{aligned}$$

39 $$\begin{aligned}&\approx \frac{(x+\beta )\sqrt{\frac{2\pi }{x+\beta }} \left( \frac{x+\beta }{e}\right) ^{x+\beta }}{(x+\alpha ) \sqrt{\frac{2\pi }{x+\alpha }}\left( \frac{x+\alpha }{e}\right) ^{x+\alpha }} \end{aligned}$$

40 $$\begin{aligned}&= \frac{\sqrt{2\pi (x+\beta )}\left( \frac{x+\beta }{e}\right) ^{x+\beta }}{\sqrt{2\pi (x+\alpha )}\left( \frac{x+\alpha }{e}\right) ^{x+\alpha }}\end{aligned}$$

41 $$\begin{aligned}&= \left( \frac{x+\beta }{x+\alpha }\right) ^{\frac{1}{2}} \frac{(x+\beta )^{x+\beta }}{(x+\alpha )^{x+\alpha }}\frac{1}{e^{\beta -\alpha }} \end{aligned}$$

42 $$\begin{aligned}&= \frac{(x+\beta )^{x+\beta +1/2}}{(x+\alpha )^{x+\alpha +1/2}} \frac{1}{e^{\beta -\alpha }} \end{aligned}$$

43 $$\begin{aligned}&= \frac{(x+\alpha +\beta -\alpha )^{x+\alpha +1/2}}{(x+\alpha )^{x+\alpha +1/2}} \frac{(x+\beta )^{\beta -\alpha }}{e^{\beta -\alpha }} \end{aligned}$$

44 $$\begin{aligned}&= \left( 1+\frac{\beta -\alpha }{x+\alpha }\right) ^{x+\alpha +1/2} \left( \frac{x+\beta }{x}\right) ^{\beta -\alpha } \frac{x^{\beta -\alpha }}{e^{\beta -\alpha }} \end{aligned}$$

45 $$\begin{aligned}&= \left( 1+\frac{\beta -\alpha }{x+\alpha }\right) ^{x+\alpha +1/2} \left( 1+\frac{\beta }{x}\right) ^{\beta -\alpha } \left( \frac{x}{e}\right) ^{\beta -\alpha }. \end{aligned}$$

We observe here that $\left( 1+\frac{\beta -\alpha }{x+\alpha }\right) ^{x+\alpha +1/2} \rightarrow e^{\beta -\alpha }$ as $x \rightarrow \infty$ and $\left( 1+\frac{\beta }{x}\right) ^{\beta -\alpha } \rightarrow 1$ as $x \rightarrow \infty$. Therefore,

46 $$\begin{aligned} \frac{\varGamma (x+\beta +1)}{\varGamma (x+\alpha +1)}&\approx e^{\beta -\alpha }\left( \frac{x}{e}\right) ^{\beta -\alpha } \end{aligned}$$

47 $$\begin{aligned}&= x^{\beta -\alpha }. \end{aligned}$$

$$\square $$

Recall that $q_{n}(i,\beta )=\frac{1}{a_{n}(\beta )} \frac{\varGamma (\beta +i+1)\varGamma (\beta +n-i+1)}{\varGamma (i+1)\varGamma (n-i+1)}$. Then, we apply Eq. 47 for large *n* and *i*,

48 $$\begin{aligned} q_{n}(i,\beta )&= \frac{1}{a_{n}(\beta )}\frac{\varGamma (\beta +i+1)}{\varGamma (i+1)} \frac{\varGamma (\beta +n-i+1)}{\varGamma (n-i+1)} \end{aligned}$$

49 $$\begin{aligned}&\approx \frac{1}{a_{n}(\beta )} i^{\beta }(n-i)^{\beta } \end{aligned}$$

50 $$\begin{aligned}&= \frac{n^{2\beta }}{a_{n}(\beta )}\left( \frac{i}{n}\right) ^{\beta } \left( 1-\frac{i}{n}\right) ^{\beta }. \end{aligned}$$

That is, $q_{n}(i,\beta )=\hat{q}_{n}(i,\beta )$ if and only if $\hat{a}_{n}(\beta )=\frac{a_{n}(\beta )}{n^{2\beta }}$. $\square$

To verify the result, we conduct a simulation for $n=500$ and $\beta =-1$ (see Fig. 13).

### Expression of First and Second Moments from Coxian PH Distribution

In this section, we derive the expressions for first and second moments from a Coxian PH distribution, then we also derive those expressions for the two examples of a Coxian PH distribution used on this paper. The structure of the rate matrix $\mathbf {Q}$ follows canonical form 3 described in Okamura and Dohi (2016).

Consider a Coxian PH distribution with four non-absorbing states defined by its rate matrix given as follows

51 $$\begin{aligned} \mathbf {Q}=\begin{bmatrix} -\lambda _{1} &{} p_{1}\lambda _{1} &{} &{} &{} \\ &{} -\lambda _{2} &{} p_{2}\lambda _{2} &{} &{} \\ &{} &{} -\lambda _{3} &{} p_{3}\lambda _{3} \\ &{} &{} &{} &{} -\lambda _{4} \end{bmatrix}, \end{aligned}$$

where $0<p_{1},p_{2},p_{3}\le 1$. Furthermore, we have the condition that $\lambda _1 \ge \lambda _{2} \ge \lambda _{3} \ge \lambda _{4}$ based on the result in Cumani (1982) and Dehon and Latouche (1982) for acyclic PH distributions.

In order to derive the expression of first and second moments of a Coxian PH distribution, we compute the inverse matrix in Eq. 51 using the identity matrix of the same size and performing elementary row operations to derive $\left( \mathbf {I}|\mathbf {(Q)^{-1}}\right)$ from $\left( \mathbf {Q}|\mathbf {I}\right)$.

$$\begin{aligned} \left( \begin{array}{rrrr|rrrr} -\lambda _{1} &{} p_{1}\lambda _{1} &{} 0 &{} 0 &{} 1 &{} 0 &{} 0 &{} 0\\ -0 &{} -\lambda _{2} &{} p_{2}\lambda _{2} &{} 0 &{} 0 &{} 1 &{} 0 &{} 0 \\ 0 &{} 0 &{} -\lambda _{3} &{} p_{3}\lambda _{3} &{} 0 &{} 0 &{} 1 &{} 0 \\ 0 &{} 0 &{} 0 &{} -\lambda _{4} &{} 0 &{} 0 &{} 0 &{} 1 \end{array}\right)&\begin{array}{l} \xrightarrow {-\frac{1}{\lambda _{1}}r_1}\\ \xrightarrow {-\frac{1}{\lambda _{2}}r_2} \end{array} \left( \begin{array}{rrrr|rrrr} 1 &{} -p_{1} &{} 0 &{} 0 &{} -\frac{1}{\lambda _{1}} &{} 0 &{} 0 &{} 0 \\ 0 &{} 1 &{} -p_{2} &{} 0 &{} 0 &{} -\frac{1}{\lambda _{2}} &{} 0 &{} 0 \\ 0 &{} 0 &{} -\lambda _{3} &{} p_{3}\lambda _{3} &{} 0 &{} 0 &{} 1 &{} 0 \\ 0 &{} 0 &{} 0 &{} -\lambda _{4} &{} 0 &{} 0 &{} 0 &{} 1 \end{array}\right) \\&\begin{array}{l} \xrightarrow {r_1+p_1r_2}\\ \xrightarrow {-\frac{1}{\lambda _{3}}r_3} \end{array} \left( \begin{array}{rrrr|rrrr} 1 &{} 0 &{} -p_{1}p_{2} &{} 0 &{} -\frac{1}{\lambda _{1}} &{} -\frac{p_{1}}{\lambda _{2}} &{} 0 &{} 0 \\ 0 &{} 1 &{} -p_{2} &{} 0 &{} 0 &{} -\frac{1}{\lambda _{2}} &{} 0 &{} 0\\ 0 &{} 0 &{} 1 &{} -p_{3} &{} 0 &{} 0 &{} -\frac{1}{\lambda _{3}} &{} 0 \\ 0 &{} 0 &{} 0 &{} -\lambda _{4} &{} 0 &{} 0 &{} 0 &{} 1 \end{array}\right) \\&\begin{array}{l} \xrightarrow {r_1+p_1p_2r_3}\\ \xrightarrow {r_2+p_2r_3} \end{array} \left( \begin{array}{rrrr|rrrr} 1 &{} 0 &{} 0 &{} -p_{1}p_{2}p_{3} &{} -\frac{1}{\lambda _{1}} &{} -\frac{p_{1}}{\lambda _{2}} &{} -\frac{p_{1}p_{2}}{\lambda _{3}} &{} 0 \\ 0 &{} 1 &{} 0 &{} -p_{2}p_{3} &{} 0 &{} -\frac{1}{\lambda _{2}} &{} -\frac{p_{2}}{\lambda _{3}} &{} 0 \\ 0 &{} 0 &{} 1 &{} -p_{3} &{} 0 &{} 0 &{} -\frac{1}{\lambda _{3}} &{} 0 \\ 0 &{} 0 &{} 0 &{} -\lambda _{4} &{} 0 &{} 0 &{} 0 &{} 1 \end{array}\right) \\&\xrightarrow {-\frac{1}{\lambda _{4}}r_4} \left( \begin{array}{rrrr|rrrr} 1 &{} 0 &{} 0 &{} -p_{1}p_{2}p_{3} &{} -\frac{1}{\lambda _{1}} &{} -\frac{p_{1}}{\lambda _{2}} &{} -\frac{p_{1}p_{2}}{\lambda _{3}} &{} 0 \\ 0 &{} 1 &{} 0 &{} -p_{2}p_{3} &{} 0 &{} -\frac{1}{\lambda _{2}} &{} -\frac{p_{2}}{\lambda _{3}} &{} 0 \\ 0 &{} 0 &{} 1 &{} -p_{3} &{} 0 &{} 0 &{} -\frac{1}{\lambda _{3}} &{} 0 \\ 0 &{} 0 &{} 0 &{} 1 &{} 0 &{} 0 &{} 0 &{} -\frac{1}{\lambda _{4}} \end{array}\right) \\&\begin{array}{l} \xrightarrow {r_1+p_1p_2p_3r_4}\\ \xrightarrow {r_2+p_2p_3r_4}\\ \xrightarrow {r_3+p_3r_4}\\ \end{array} \left( \begin{array}{rrrr|rrrr} 1 &{} 0 &{} 0 &{} 0 &{} -\frac{1}{\lambda _{1}} &{} -\frac{p_{1}}{\lambda _{2}} &{} -\frac{p_{1}p_{2}}{\lambda _{3}} &{} -\frac{p_{1}p_{2}p_{3}}{\lambda _{4}} \\ 0 &{} 1 &{} 0 &{} 0 &{} 0 &{} -\frac{1}{\lambda _{2}} &{} -\frac{p_{2}}{\lambda _{3}} &{} -\frac{p_{2}p_{3}}{\lambda _{4}} \\ 0 &{} 0 &{} 1 &{} 0 &{} 0 &{} 0 &{} -\frac{1}{\lambda _{3}} &{} -\frac{p_{3}}{\lambda _{4}} \\ 0 &{} 0 &{} 0 &{} 1 &{} 0 &{} 0 &{} 0 &{} -\frac{1}{\lambda _{4}} \end{array}\right) . \end{aligned}$$

Therefore,

$$\begin{aligned}&\mathbf {Q}^{-1}=\begin{bmatrix} -\frac{1}{\lambda _{1}} &{} -\frac{p_{1}}{\lambda _{2}} &{} -\frac{p_{1}p_{2}}{\lambda _{3}} &{} -\frac{p_{1}p_{2}p_{3}}{\lambda _{4}} \\ 0 &{} -\frac{1}{\lambda _{2}} &{} -\frac{p_{2}}{\lambda _{3}} &{} -\frac{p_{2}p_{3}}{\lambda _{4}}\\ 0 &{} 0 &{} -\frac{1}{\lambda _{3}} &{} -\frac{p_{3}}{\lambda _{4}}\\ 0 &{} 0 &{} 0 &{} -\frac{1}{\lambda _{4}} \end{bmatrix} \end{aligned}$$

and $\mathbf {Q}\mathbf {Q}^{-1}=\mathbf {I}$ where $\mathbf {I}$ is the identity matrix.

Furthermore,

$$\begin{aligned} \mathbf {Q}^{-2}&=\left( \mathbf {Q}^{-1}\right) ^2\\&=\begin{bmatrix} \frac{1}{\lambda _{1}^2} &{} \frac{p_{1}}{\lambda _{2}}\left( \frac{1}{\lambda _{1}}+\frac{1}{\lambda _{2}}\right) &{} \frac{p_{1}p_{2}}{\lambda _{3}}\left( \frac{1}{\lambda _{1}}+\frac{1}{\lambda _{2}}+\frac{1}{\lambda _{3}}\right) &{} \frac{p_{1}p_{2}p_{3}}{\lambda _{4}}\left( \frac{1}{\lambda _{1}}+\frac{1}{\lambda _{2}}+\frac{1}{\lambda _{3}}+\frac{1}{\lambda _{4}}\right) \\ 0 &{} \frac{1}{\lambda _{2}^2} &{} \frac{p_{2}}{\lambda _{3}}\left( \frac{1}{\lambda _{2}}+\frac{1}{\lambda _{3}}\right) &{} \frac{p_{2}p_{3}}{\lambda _{4}}\left( \frac{1}{\lambda _{2}}+\frac{1}{\lambda _{3}}+\frac{1}{\lambda _{4}}\right) \\ 0 &{} 0 &{} \frac{1}{\lambda _{3}^2} &{} \frac{p_{3}}{\lambda _{4}}\left( \frac{1}{\lambda _{3}}+\frac{1}{\lambda _{4}}\right) \\ 0 &{} 0 &{} 0 &{} \frac{1}{\lambda _{4}^2} \end{bmatrix}. \end{aligned}$$

Hence, the expressions for first and second moments from a Coxian PH distribution with the initial probability distribution $\varvec{\alpha }=\left[ 1, 0, 0, 0 \right]$ and the rate matrix given by Eq. 51 are as follows

52 $$\begin{aligned} {\mathbb {E}}_{\mathrm{PH}}(X)&= -\varvec{\alpha }\mathbf {Q}^{-1}\mathbf {1} \nonumber \\&=-\begin{bmatrix} 1&0&0&0 \end{bmatrix} \begin{bmatrix} -\frac{1}{\lambda _{1}} &{} -\frac{p_{1}}{\lambda _{2}} &{} -\frac{p_{1}p_{2}}{\lambda _{3}} &{} -\frac{p_{1}p_{2}p_{3}}{\lambda _{4}} \\ 0 &{} -\frac{1}{\lambda _{2}} &{} -\frac{p_{2}}{\lambda _{3}} &{} -\frac{p_{2}p_{3}}{\lambda _{4}}\\ 0 &{} 0 &{} -\frac{1}{\lambda _{3}} &{} -\frac{p_{3}}{\lambda _{4}}\\ 0 &{} 0 &{} 0 &{} -\frac{1}{\lambda _{4}} \end{bmatrix} \begin{bmatrix} 1 \\ 1 \\ 1 \\ 1 \end{bmatrix}\nonumber \\&= \frac{1}{\lambda _{1}}+\frac{p_{1}}{\lambda _{2}}+\frac{p_{1}p_{2}}{\lambda _{3}} +\frac{p_{1}p_{2}p_{3}}{\lambda _{4}}, \nonumber \\ {\mathbb {E}}_{\mathrm{PH}}(X)&=\frac{1}{\lambda _{1}}+p_{1}\left( \frac{1}{\lambda _{2}} +p_{2}\left( \frac{1}{\lambda _{3}}+\frac{p_{3}}{\lambda _{4}}\right) \right) . \end{aligned}$$

53 $$\begin{aligned} {\mathbb {E}}_{\mathrm{PH}}\left( X^{2}\right)&= 2\varvec{\alpha }\mathbf {Q}^{-2}\mathbf {1} \nonumber \\&=2\begin{bmatrix} 1&0&0&0 \end{bmatrix}\nonumber \\&\quad \begin{bmatrix} \frac{1}{\lambda _{1}^2} &{} \frac{p_{1}}{\lambda _{2}}\left( \frac{1}{\lambda _{1}}+\frac{1}{\lambda _{2}}\right) &{} \frac{p_{1}p_{2}}{\lambda _{3}}\left( \frac{1}{\lambda _{1}}+\frac{1}{\lambda _{2}}+\frac{1}{\lambda _{3}}\right) &{} \frac{p_{1}p_{2}p_{3}}{\lambda _{4}}\left( \frac{1}{\lambda _{1}}+\frac{1}{\lambda _{2}}+\frac{1}{\lambda _{3}}+\frac{1}{\lambda _{4}}\right) \\ 0 &{} \frac{1}{\lambda _{2}^2} &{} \frac{p_{2}}{\lambda _{3}}\left( \frac{1}{\lambda _{2}}+\frac{1}{\lambda _{3}}\right) &{} \frac{p_{2}p_{3}}{\lambda _{4}}\left( \frac{1}{\lambda _{2}}+\frac{1}{\lambda _{3}}+\frac{1}{\lambda _{4}}\right) \\ 0 &{} 0 &{} \frac{1}{\lambda _{3}^2} &{} \frac{p_{3}}{\lambda _{4}}\left( \frac{1}{\lambda _{3}}+\frac{1}{\lambda _{4}}\right) \\ 0 &{} 0 &{} 0 &{} \frac{1}{\lambda _{4}^2} \end{bmatrix} \begin{bmatrix} 1 \\ 1 \\ 1 \\ 1 \end{bmatrix}, \nonumber \\ {\mathbb {E}}_{\mathrm{PH}}\left( X^{2}\right)&= 2\left[ \frac{1}{\lambda _{1}^{2}}+\frac{p_{1}}{\lambda _{2}} \left( \frac{1}{\lambda _{1}}+\frac{1}{\lambda _{2}}\right) +\frac{p_{1}p_{2}}{\lambda _{3}}\left( \frac{1}{\lambda _{1}} +\frac{1}{\lambda _{2}}+\frac{1}{\lambda _{3}}\right) \right. \nonumber \\&\qquad \quad \left. +\frac{p_{1}p_{2}p_{3}}{\lambda _{4}}\left( \frac{1}{\lambda _{1}} +\frac{1}{\lambda _{2}}+\frac{1}{\lambda _{3}}+\frac{1}{\lambda _{4}}\right) \right] . \end{aligned}$$

Next, to get the expressions for first and second moment from $\mathrm{PH}_{\mathrm{Dec}}$, we use Eqs. 52 and 53 and the following substitutions,

54 $$\begin{aligned}&\lambda _{1}=z,\lambda _{2}=1+x,\lambda _{3}=1+x^{2},\lambda _{4}=x^{3}, \nonumber \\&p_{1}=1-y,p_{2}=1-y^{2},p_{3}=1-y^{3}. \end{aligned}$$

On the other hand, to derive the expressions for both moments from $\mathrm{PH}_{\mathrm{Inc}}$, we use the following substitutions to Eqs. 52 and 53,

55 $$\begin{aligned}&\lambda _{1}=1+x^{3},\lambda _{2}=1+x^{2},\lambda _{3}=1+x,\lambda _{4}=z \nonumber \\&p_{1}=1-y^{4},p_{2}=1-y^{3},p_{3}=1-y^{2} \end{aligned}$$

### Deriving and Solving the Differential Equation of $D^{(1)}_{t}(z)$

In this section, we show the derivation of the differential equation of the probability of observing a reconstructed external branch with length *z* on a tree with age *t*, $D^{(1)}_{t}(z)$, shown in Eq. 23, using physical interpretations. Then, we derive the solution to the differential equation as shown in Eq. 26.

We can write $D^{(1)}_{t}(z)$ by conditioning on the time of the first event on that external branch with elapsed time *z* on a tree with age *t*. That is, (1) the branch has not undergone any observable event yet at time *z*, which occurs with probability $e^{-(\lambda +\mu )z}$ or (2) the branch has a child at some time $u \le z$, which occurs with probability $e^{-(\lambda +\mu )u}\lambda$, and so the two branches evolve independently of each other where the child branch becomes extinct by time *z* with probability $E(z-u)$ and the initial branch survives at time *z* with probability $D^{(1)}_{t}(z-u)$ or vice versa. Thus,

$$\begin{aligned} D^{(1)}_{t}(z) = e^{-(\lambda +\mu )z} + \int _{u=0}^{z}e^{-(\lambda +\mu )u} \lambda \left( 2D^{(1)}_{t}(z-u)E(z-u)\right) \mathrm{d}u. \end{aligned}$$

Then, by taking derivative with respect to *z* in the above equation, we have,

$$\begin{aligned} \frac{\mathrm{d}D^{(1)}_{t}(z)}{\mathrm{d}z}= & {} \frac{\mathrm{d}}{\mathrm{d}z}\left( e^{-(\lambda +\mu )z}\right) +\frac{\mathrm{d}}{\mathrm{d}z}\left( \int _{u=0}^{z}e^{-(\lambda +\mu )u}\lambda \left( 2D^{(1)}_{t}(z-u)E(z-u)\right) \mathrm{d}u\right) . \end{aligned}$$

Next, by applying the Leibniz integral rule and noting that $E(z-z)=E(0)=0$, we have,

$$\begin{aligned} \frac{\mathrm{d}D^{(1)}_{t}(z)}{\mathrm{d}z}= & {} -(\lambda +\mu )e^{-(\lambda +\mu )z} +\left( \int _{u=0}^{z}\frac{\partial }{\partial z} \left( e^{-(\lambda +\mu )u}\lambda \left( 2D^{(1)}_{t}(z-u) E(z-u)\right) \right) \mathrm{d}u\right) \\= & {} -(\lambda +\mu )e^{-(\lambda +\mu )z} + \int _{u=0}^{z} e^{-(\lambda +\mu )u}2\lambda \\&\left( \frac{\partial D^{(1)}_{t}(z-u)}{\partial z} E(z-u)+D^{(1)}_{t}(z-u)\frac{\partial E(z-u)}{\partial z}\right) \mathrm{d}u. \end{aligned}$$

Next, applying integration by parts and noting:

$$\begin{aligned} \frac{\partial }{\partial u}(D^{(1)}_{t}(z-u)E(z-u)) = -\left( \frac{\partial D^{(1)}_{t}(z-u)}{\partial z} E(z-u)+D^{(1)}_{t}(z-u)\frac{\partial E(z-u)}{\partial z}\right) \end{aligned}$$

we get,

$$\begin{aligned} \frac{\mathrm{d}D^{(1)}_{t}(z)}{\mathrm{d}z}= & {} -(\lambda +\mu )e^{-(\lambda +\mu )z} \nonumber \\&+ \left( -2\lambda e^{-(\lambda +\mu )u} D^{(1)}_{t}(z-u)E(z-u)\vert _{u=0}^{z}\right. \nonumber \\&\left. -\int _{u=0}^{z}2\lambda (\lambda +\mu )e^{-(\lambda +\mu )u} D^{(1)}_{t}(z-u)E(z-u)\mathrm{d}u\right) \nonumber \\= & {} -(\lambda +\mu )e^{-(\lambda +\mu )z} + 2\lambda D^{(1)}_{t}(z) E(z)-(\lambda + \mu )\nonumber \\&\int _{u=0}^{z}2\lambda e^{-(\lambda +\mu )u} D^{(1)}_{t}(z-u)E(z-u)\mathrm{d}u \nonumber \\= & {} -(\lambda +\mu )\left( e^{-(\lambda +\mu )z}+\int _{u=0}^{z} e^{-(\lambda +\mu )u}\lambda \left( 2D^{(1)}_{t}(z-u)E(z-u)\right) \mathrm{d}u\right) \nonumber \\&+ 2\lambda D^{(1)}_{t}(z)E(z)\nonumber \\ \frac{\mathrm{d}D^{(1)}_{t}(z)}{\mathrm{d}z}= & {} -(\lambda +\mu )D^{(1)}_{t}(z) + 2\lambda E(z)D^{(1)}_{t}(z), \quad \text {as in Eq}.~(23) \end{aligned}$$

#### Lemma 3

$$\begin{aligned} D^{(1)}_{t}(z)=\left( \frac{(\lambda -\mu )e^{\mu z}}{\lambda -\mu e^{(\mu -\lambda )z}}\right) ^{2}e^{-(\lambda +\mu )z} \end{aligned}$$

is the solution to the differential equation,

$$\begin{aligned} \frac{\mathrm{d}D^{(1)}_{t}(z)}{\mathrm{d} z}= & {} -(\lambda +\mu )D^{(1)}_{t}(z) + 2\lambda E(z)D^{(1)}_{t}(z), \end{aligned}$$

where $$D^{(1)}_{t}(0)=1$$.

#### Proof

Substitute *E*(*z*) from Eq. (24) (see also Kendall 1948) to the above differential equation, we have,

56 $$\begin{aligned} \frac{\mathrm{d}D^{(1)}_{t}(z)}{\mathrm{d}z} = -(\lambda + \mu )D^{(1)}_{t}(z) +2\lambda \left( \frac{\mu - \mu e^{(\mu -\lambda )z}}{\lambda - \mu e^{(\mu -\lambda )z}}\right) D^{(1)}_{t}(z). \end{aligned}$$

Then,

57 $$\begin{aligned} \frac{\mathrm{d}D^{(1)}_{t}(z)}{\mathrm{d}z}= & {} -(\lambda + \mu )D^{(1)}_{t}(z) + 2\lambda \left( \frac{\mu - \mu e^{(\mu -\lambda )z}}{\lambda - \mu e^{(\mu -\lambda )z}}\right) D^{(1)}_{t}(z) \nonumber \\ \frac{\mathrm{d}D^{(1)}_{t}(z)}{\mathrm{d}z}= & {} -(\lambda + \mu )D^{(1)}_{t}(z) + g(z)D^{(1)}_{t}(z), \quad g(z) = 2\lambda \left( \frac{\mu - \mu e^{(\mu -\lambda )z}}{\lambda - \mu e^{(\mu -\lambda )z}}\right) \nonumber \\ \frac{\mathrm{d}D^{(1)}_{t}(z)}{\mathrm{d}z}= & {} \left( g(z)-(\lambda + \mu )\right) D^{(1)}_{t}(z) \nonumber \\ \int {\frac{dD^{(1)}_{t}(z)}{D^{(1)}_{t}(z)}}= & {} \int {\left( g(z) -(\lambda + \mu )\right) }\mathrm{d}z + C \nonumber \\ \ln {\left( D^{(1)}_{t}(z)\right) }= & {} -(\lambda + \mu )z + \int {g(z)\mathrm{d}z} + C. \end{aligned}$$

Next, we solve $\int {g(z)\mathrm{d}z}$,

$$\begin{aligned} \int {g(z)\mathrm{d}z}= & {} 2\lambda \int {\frac{\mu - \mu e^{(\mu -\lambda )z}}{\lambda - \mu e^{(\mu -\lambda )z}}}\mathrm{d}z \\= & {} 2\lambda \left( \underbrace{\int {\frac{\mu \mathrm{d}z}{\lambda - \mu e^{(\mu -\lambda )z}}}}_{I}-\underbrace{\int {\frac{\mu e^{(\mu -\lambda )z} \mathrm{d}z}{\lambda - \mu e^{(\mu -\lambda )z}}}}_{II}\right) . \end{aligned}$$

We solve the integrals in *I* and *II*,

$$\begin{aligned} I= & {} \int {\frac{\mu \mathrm{d}z}{\lambda - \mu e^{(\mu -\lambda )z}}} \\= & {} \frac{\mu }{\mu -\lambda }\int {\frac{\mathrm{d}v}{v(\lambda - v)}}, \quad v = \mu e^{(\mu -\lambda )z}, \quad dv = v(\mu -\lambda )dz \\= & {} \frac{\mu }{\mu -\lambda } \left[ \int {\frac{\mathrm{d}v}{\lambda v}} + \int {\frac{\mathrm{d}v}{\lambda (\lambda - v)}}\right] \\= & {} \frac{\mu }{\mu -\lambda } \left[ \frac{1}{\lambda }\ln {v} - \frac{1}{\lambda } \ln {(\lambda -v)}+c_{0}\right] \\= & {} \frac{\mu }{\lambda (\mu -\lambda )} \left[ \ln {\left( \frac{v}{\lambda -v}\right) }+c_{0}\right] \\ I= & {} \frac{\mu }{\lambda (\mu -\lambda )} \ln {\left( \frac{\mu e^{(\mu -\lambda )z}}{\lambda - \mu e^{(\mu -\lambda )z}}\right) }+c_{1}, \end{aligned}$$

and

$$\begin{aligned} II= & {} \int {\frac{\mu e^{(\mu -\lambda )z} \mathrm{d}z}{\lambda - \mu e^{(\mu -\lambda )z}}}\\= & {} \int {\frac{m\mathrm{d}m}{(\mu -\lambda )m(\lambda - m)}}, \quad m=\mu e^{(\mu -\lambda )z}, \quad \mathrm{d}m=(\mu -\lambda )m\mathrm{d}z\\= & {} \frac{1}{\mu -\lambda }\int {\frac{\mathrm{d}m}{\lambda -m}}\\= & {} \frac{1}{\lambda - \mu }\ln {(\lambda - m)} + c\\ II= & {} \frac{1}{\lambda - \mu }\ln {\left( \lambda -\mu e^{(\mu -\lambda )z}\right) } + c. \end{aligned}$$

Thus,

$$\begin{aligned} \int {g(z)\mathrm{d}z}= & {} 2\lambda \left( \frac{\mu }{\lambda (\mu -\lambda )} \ln {\left( \frac{\mu e^{(\mu -\lambda )z}}{\lambda - \mu e^{(\mu -\lambda )z}}\right) } -\frac{1}{\lambda - \mu }\ln {\left( \lambda -\mu e^{(\mu -\lambda )z}\right) }\right) +c\\= & {} 2\lambda \left( \frac{\mu }{\lambda (\mu -\lambda )} \ln {\left( \mu e^{(\mu -\lambda )z}\right) }+\frac{\lambda -\mu }{\lambda (\mu -\lambda )} \ln {\left( \lambda -\mu e^{(\mu -\lambda )z}\right) }\right) +c\\= & {} 2\left( \frac{\mu }{\mu -\lambda }\ln {\left( \mu e^{(\mu -\lambda )z}\right) } -\ln {\left( \lambda -\mu e^{(\mu -\lambda )z}\right) }\right) +c. \end{aligned}$$

Substituting back into Eq. (58), we get,

$$\begin{aligned} \ln {\left( D^{(1)}_{t}(z)\right) }= & {} -(\lambda + \mu )z + 2\left( \frac{\mu }{\mu -\lambda }\ln {\left( \mu e^{(\mu -\lambda )z}\right) } -\ln {\left( \lambda -\mu e^{(\mu -\lambda )z}\right) }\right) + C \nonumber \\ \ln {\left( D^{(1)}_{t}(z)\right) }= & {} -(\lambda + \mu )z + 2 \ln {\left( \frac{\left( \mu e^{(\mu -\lambda )z}\right) ^{\frac{\mu }{\mu -\lambda }}}{\left( \lambda -\mu e^{(\mu -\lambda )z}\right) }\right) } + C \nonumber \\ D^{(1)}_{t}(z)= & {} K\left( \frac{\left( \mu e^{(\mu -\lambda )z}\right) ^{\frac{\mu }{\mu -\lambda }}}{\left( \lambda -\mu e^{(\mu -\lambda )z}\right) }\right) ^{2} e^{-(\lambda +\mu )z}. \end{aligned}$$

Since $D^{(1)}_{t}(0)=1$, we have

$$\begin{aligned} K = \left( \frac{\lambda -\mu }{\mu ^{\frac{\mu }{\mu -\lambda }}}\right) ^{2}. \end{aligned}$$

Therefore,

$$\begin{aligned} D^{(1)}_{t}(z)= & {} \left[ \frac{\lambda -\mu }{\mu ^{\frac{\mu }{\mu -\lambda }}} \times \frac{(\mu e^{(\mu -\lambda )z})^{\frac{\mu }{\mu -\lambda }}}{(\lambda -\mu e^{(\mu -\lambda )z})}\right] ^{2} e^{-(\lambda +\mu )z} \nonumber \\= & {} \left[ \frac{(\lambda -\mu )e^{\mu z}}{\lambda - \mu e^{(\mu -\lambda )z}}\right] ^{2} e^{-(\lambda +\mu )z}. \end{aligned}$$

$$\square $$

### Deriving and Solving the Differential Equation of $G_{x,t}(z)$

Here, we show the derivation of the differential equation of the probability of observing a reconstructed internal branch with length *z* on a tree with age *t*, $G_{x,t}(z)$, shown in Eq. 21, using physical interpretations where *x* is the length of an external branch descending from that internal branch. Then, we derive the solution to the differential equation as shown in Eq. 25.

We can write $G_{x,t}(z)$ by conditioning on the time of the first event on that internal branch with elapsed time *z* on a tree with age *t* (the elapsed time since the beginning of the tree starting at time 0). That is, (1) the branch has not undergone any observable event yet at time $z \le t$, which occurs with probability $e^{-(\lambda +\mu )z}$ or (2) the branch has a child at some time $z-u \le z$, which occurs with probability $e^{-(\lambda +\mu )(z-u)}\lambda$, and so the two branches evolve independently of each other where the child branch becomes extinct by time *t* with elapsed time $u+x$ with probability $E(u+x)$ and the initial branch survives until time $u \le z$ with probability $G_{x,t}(u)$ or vice versa. Thus,

$$\begin{aligned} G_{x,t}(z) = e^{-(\lambda +\mu )z} + \int _{u=0}^{z}e^{-(\lambda +\mu )(z-u)} \lambda \left( 2G_{x,t}(u)E(u+x)\right) \mathrm{d}u. \end{aligned}$$

Taking the derivative with respect to *z* on both sides of the equation above, we have

$$\begin{aligned} \frac{\mathrm{d}G_{x,t}(z)}{\mathrm{d}z}= & {} \frac{\mathrm{d}}{\mathrm{d}z}\left( e^{-(\lambda +\mu )z}\right) +\frac{\mathrm{d}}{\mathrm{d}z}\left( \int _{u=0}^{z}e^{-(\lambda +\mu )(z-u)}\lambda \left( 2G_{x,t}(u)E(u+x)\right) \mathrm{d}u\right) . \end{aligned}$$

Then, by applying the Leibniz integral rule we have,

$$\begin{aligned} \frac{\mathrm{d}G_{x,t}(z)}{\mathrm{d}z}= & {} -(\lambda +\mu )e^{-(\lambda +\mu )z} + 2\lambda G_{x,t}(z)E(z+x) \nonumber \\&+ \int _{u=0}^{z}\frac{\mathrm{d}}{\mathrm{d}z} \left( e^{-(\lambda +\mu )(z-u)}\lambda (2G_{x,t}(u)E(u+x))\right) \mathrm{d}u\nonumber \\= & {} -(\lambda +\mu )e^{-(\lambda +\mu )z}+ 2\lambda G_{x,t}(z)E(z+x) -(\lambda +\mu )\nonumber \\&\int _{u=0}^{z}e^{-(\lambda +\mu )(z-u)}\lambda 2 G_{x,t}(u)E(u+x) \mathrm{d}u\nonumber \\= & {} -(\lambda +\mu ) \left( e^{-(\lambda +\mu )z}+\int _{u=0}^{z} e^{-(\lambda +\mu )(z-u)}\lambda 2 G_{x,t}(u)E(u+x) \mathrm{d}u\right) \nonumber \\&+2\lambda G_{x,t}(z)E(z+x)\nonumber \\= & {} -(\lambda +\mu )G_{x,t}(z) + 2\lambda G_{x,t}(z)E(z+x), \quad \text {as in Eq}.~21. \end{aligned}$$

#### Lemma 4

$$\begin{aligned} G_{x,t}(z)= \left( \frac{\lambda -\mu e^{(\mu -\lambda )x}}{\lambda - \mu e^{(\mu -\lambda )(z+x)}}\right) ^{2}e^{(\mu -\lambda )z} \end{aligned}$$

is the solution to the differential equation,

$$\begin{aligned} \frac{\mathrm{d}G_{x,t}(z)}{\mathrm{d}z} = -(\lambda +\mu )G_{x,t}(z) + 2\lambda G_{x,t}(z)E(z+x), \end{aligned}$$

where $$G_{x,t}(0)=1$$.

#### Proof

We solve the differential equation for $G_{x,t}(z)$ as follows

58 $$\begin{aligned} \frac{\mathrm{d}G_{x,t}(z)}{\mathrm{d}z}= & {} -(\lambda +\mu )G_{x,t}(z) + 2\lambda G_{x,t}(z)E(z+x)\nonumber \\ \int {\frac{dG_{x,t}(z)}{G_{x,t}(z)}}= & {} \int {\left( -(\lambda +\mu )+2 \lambda E(z+x)\right) \mathrm{d}z}+C\nonumber \\ \ln {\left( G_{x,t}(z)\right) }= & {} -(\lambda +\mu )z+2\lambda \int {E(z+x)\mathrm{d}z}+C\nonumber \\ \ln {\left( G_{x,t}(z)\right) }= & {} -(\lambda +\mu )z+2\lambda \int {E(v)\mathrm{d}v}+C, \quad v =z +x, \quad dv = dz\nonumber \\ G_{x,t}(z)= & {} K e^{-(\lambda +\mu )z + 2\lambda \int {E(v)\mathrm{d}v}}. \end{aligned}$$

Next, we solve $\int {E(v)\mathrm{d}v}$.

$$\begin{aligned} \int {E(v)\mathrm{d}v}= & {} \int {\frac{\mu -\mu e^{-(\lambda -\mu )v}}{\lambda -\mu e^{-(\lambda -\mu )v}}\mathrm{d}v}\\= & {} \int {\frac{\mu -w}{-(\lambda -w)}\frac{1}{(\lambda -\mu )w}\mathrm{d}w}, \quad w=\mu e^{-(\lambda -\mu )v}, \quad \mathrm{d}w=-(\lambda -\mu )w \mathrm{d}\mathrm{d}v\\= & {} \frac{1}{\lambda -\mu }\int {\frac{w-\mu }{(\lambda -w)w}\mathrm{d}w}\\= & {} \frac{1}{\lambda -\mu } \left( \int {\frac{1}{\lambda -w}\mathrm{d}w} -\int {\frac{\mu }{(\lambda -w)w}\mathrm{d}w}\right) \\= & {} \frac{1}{\lambda -\mu } (A-B). \end{aligned}$$

Note that

$$\begin{aligned} A= & {} \int {\frac{1}{\lambda -w}\mathrm{d}w} \\= & {} -\int {\frac{\mathrm{d}(\lambda -w)}{\lambda -w}} \\= & {} -\ln {(\lambda -w)}+C.\\ B= & {} \int {\frac{\mu }{(\lambda -w)w}\mathrm{d}w} \\= & {} \mu \int {\frac{1}{(\lambda -w)w}\mathrm{d}w}\\= & {} \mu \left( \int {\frac{1}{\lambda (\lambda -w)}\mathrm{d}w +\int {\frac{1}{\lambda w}\mathrm{d}w}}\right) \\= & {} \mu \left( -\frac{1}{\lambda }\ln {(\lambda -w)}+\frac{1}{\lambda }\ln (w)\right) \\= & {} \frac{\mu }{\lambda }\left( \ln (w)-\ln (\lambda -w)\right) \\= & {} \ln {\left( \frac{w}{\lambda -w}\right) ^{\frac{\mu }{\lambda }}}+C.\\ \end{aligned}$$

Thus,

$$\begin{aligned} \int {E(v)}\mathrm{d}v= & {} \frac{1}{\lambda -\mu }\left( -\ln {(\lambda -w)} -\ln {\left( \frac{w}{\lambda -w}\right) ^{\frac{\mu }{\lambda }}}\right) \\= & {} \frac{1}{\mu -\lambda } \left( \ln \left( (\lambda -w) \frac{w^{\frac{\mu }{\lambda }}}{(\lambda -w)^{\frac{\mu }{\lambda }}}\right) \right) \\= & {} \frac{1}{\mu -\lambda } \left( \ln \left( \frac{w^{\frac{\mu }{\lambda }}}{(\lambda -w)^{\frac{\mu }{\lambda }-1}}\right) \right) \\= & {} \ln \left( \frac{w^{\frac{\mu }{\lambda }}}{(\lambda -w)^{\frac{\mu }{\lambda }-1}}\right) ^{\frac{1}{\mu -\lambda }}\\= & {} \ln \left( \frac{(\mu e^{-(\lambda -\mu )v})^{\frac{\mu }{\lambda }}}{(\lambda -\mu e^{-(\lambda -\mu )v})^{\frac{\mu }{\lambda }-1}} \right) ^{\frac{1}{\mu -\lambda }}. \end{aligned}$$

Substituting this back into Eq. (58), we get,

$$\begin{aligned} G_{x,t}(z)= & {} K e^{-(\lambda +\mu )z + 2\lambda \int {E(v)\mathrm{d}v}} \\= & {} K \left( e^{-(\lambda +\mu )z} \left( \frac{(\mu e^{-(\lambda -\mu )v})^{\frac{\mu }{\lambda }}}{(\lambda -\mu e^{-(\lambda -\mu ) v})^{\frac{\mu }{\lambda }-1}}\right) ^{\frac{2\lambda }{\mu -\lambda }}\right) \\= & {} K \left( e^{-(\lambda +\mu )z} \frac{\left( \mu e^{-(\lambda -\mu ) v}\right) ^{\frac{2\mu }{\mu -\lambda }}}{\left( \lambda -\mu e^{-(\lambda -\mu )v}\right) ^{2}}\right) \\= & {} K \left( \frac{\left( \mu e^{-(\lambda -\mu )v}\right) ^{\frac{\mu }{\mu -\lambda }}}{\lambda -\mu e^{-(\lambda -\mu )v}}\right) ^{2}e^{-(\lambda +\mu )z}\\= & {} K \left( \frac{\mu ^{\frac{\mu }{\mu -\lambda }}e^{\mu v}}{\lambda -\mu e^{-(\lambda -\mu )v}}\right) ^{2}e^{-(\lambda +\mu )z}\\= & {} K \left( \frac{\mu ^{\frac{\mu }{\mu -\lambda }}e^{\mu (z+x)}}{\lambda -\mu e^{(\mu -\lambda )(z+x)}}\right) ^{2}e^{-(\lambda +\mu )z}. \end{aligned}$$

Given that $G_{x,t}(0)=1$, we have,

$$\begin{aligned} K = \left( \frac{\lambda -\mu e^{(\mu -\lambda )x}}{\mu ^{\frac{\mu }{\mu -\lambda }} e^{\mu x}}\right) ^{2}. \end{aligned}$$

Thus,

59 $$\begin{aligned} G_{x,t}(z)= & {} \left( \frac{\lambda -\mu e^{(\mu -\lambda )x}}{\mu ^{\frac{\mu }{\mu -\lambda }}e^{\mu x}}\right) ^{2} \left( \frac{\mu ^{\frac{\mu }{\mu -\lambda }}e^{\mu (z+x)}}{\lambda -\mu e^{(\mu -\lambda )(z+x)}}\right) ^{2}e^{-(\lambda +\mu )z}\nonumber \\= & {} \frac{\left( \lambda -\mu e^{(\mu -\lambda )x}\right) ^{2} e^{(\mu -\lambda )z}}{\left( \lambda -\mu e^{(\mu -\lambda )(z+x)}\right) ^{2}}\nonumber \\= & {} \left( \frac{\lambda -\mu e^{(\mu -\lambda )x}}{\lambda -\mu e^{(\mu -\lambda )(z+x)}}\right) ^{2}e^{(\mu -\lambda )z}. \end{aligned}$$

$$\square $$

### Showing the Relationship with ${\mathcal {L}}_{\mathrm{Nee}}(T^* \ |\ \lambda , \mu )$

Here, we show the relationship between the likelihood ${\mathcal {L}}(T^* \ |\ \lambda , \mu )$ shown in Eq. 19 with the likelihood ${\mathcal {L}}_{\mathrm{Nee}}(T^* \ |\ \lambda , \mu )$ in Nee et al. (1994b). We show this for the case of the reconstructed tree $T^*$ with three tips as pictured in Fig. 3. On this reconstructed tree with age *t*, ignoring the age of the root, the external branch 2 is one of the two original branches descending from the root, so it has length $\tilde{b}_2=x_2$. We assume that the other two external branches have length $\tilde{b}_3=\tilde{b}_4=x_3$. Thus, the internal branch 1 has length $b_1=x_2-x_3$.

#### Lemma 5

$$\begin{aligned} {\mathcal {L}}_{\mathrm{Nee}}(T^* \ |\ \lambda , \mu ) = \frac{{\mathcal {L}}(T^* \ |\ \lambda , \mu )}{\left( 1-E(x_{2})\right) ^{2}}, \end{aligned}$$

where ${\mathcal {L}}(T^* \ |\ \lambda , \mu )$ is given in Eq. 19, ${\mathcal {L}}_{\mathrm{Nee}}(T^* \ |\ \lambda , \mu )$ is given in Eq. (20) in Nee et al. (1994b), $x_2$ is the elapsed time from the starting time of the two original branches descending from the root of the tree $T^*$ until the end of the tree, and $E(x_{2})$ is the extinction probability for one of those branches.

#### Proof

Under the reconstructed tree $T^*$ with three tips, the likelihood in Eq. 19 is simplified to,

$$\begin{aligned} {\mathcal {L}}(T^* \ |\ \lambda , \mu )= & {} 2!\times G_{x_1,t}(b_1)\times \lambda \times D^{(1)}_{t}(\tilde{b}_2) \times D^{(1)}_{t}(\tilde{b}_3)\times D^{(1)}_{t}(\tilde{b}_4)\\= & {} 2\lambda \left( \frac{\lambda -\mu e^{-(\lambda -\mu )x_3}}{\lambda -\mu e^{-(\lambda -\mu )x_2}}\right) ^{2}e^{-(\lambda -\mu )(x_2-x_3)} \left( \frac{(\lambda -\mu ) e^{\mu x_2}}{\lambda -\mu e^{-(\lambda -\mu )x_2}}\right) ^{2} e^{-(\lambda +\mu )x_2}\\&\left( \frac{(\lambda -\mu ) e^{\mu x_3}}{\lambda -\mu e^{-(\lambda -\mu )x_3}}\right) ^{4} e^{-2(\lambda +\mu )x_3}\\= & {} 2\lambda \left( \frac{1}{\lambda -\mu e^{-(\lambda -\mu )x_2}}\right) ^{2} \left( \frac{(\lambda -\mu ) e^{-(\lambda -\mu )x_2}}{\lambda -\mu e^{-(\lambda -\mu )x_2}}\right) ^{2}\\&\left( \frac{(\lambda -\mu ) e^{-(\lambda -\mu )x_3}}{\lambda -\mu e^{-(\lambda -\mu )x_3}}\right) ^{2} \left( \frac{(\lambda -\mu )^3}{\lambda -\mu e^{-(\lambda -\mu )x_3}}\right) \\= & {} 2\lambda \left( \frac{\lambda -\mu }{\lambda -\mu e^{-(\lambda -\mu )x_3}}\right) \left( \frac{(\lambda -\mu )e^{-(\lambda -\mu )x_2}}{\lambda -\mu e^{-(\lambda -\mu )x_2}}\right) ^{2}\\&\left( \frac{(\lambda -\mu )e^{-(\lambda -\mu )x_3}}{\lambda -\mu e^{-(\lambda -\mu )x_3}}\right) \left( \frac{\lambda -\mu }{\lambda -\mu e^{-(\lambda -\mu )x_2}}\right) ^{2}\\= & {} {\mathcal {L}}_{\mathrm{Nee}}(T^* \ |\ \lambda , \mu ){\left( 1-E(x_{2})\right) ^{2}}\\&{\mathcal {L}}_{\mathrm{Nee}}(T^* \ |\ \lambda , \mu ) = \frac{{\mathcal {L}} (T^* \ |\ \lambda , \mu )}{\left( 1-E(x_{2})\right) ^{2}}. \end{aligned}$$

$$\square $$
